# Terror Management in a Multicultural Society: Effects of Mortality Salience on Attitudes to Multiculturalism Are Moderated by National Identification and Self-Esteem Among Native Dutch People

**DOI:** 10.3389/fpsyg.2018.00721

**Published:** 2018-05-15

**Authors:** Mandy Tjew-A-Sin, Sander Leon Koole

**Affiliations:** Department of Clinical, Neuro- and Developmental Psychology, Vrije Universiteit Amsterdam, Amsterdam, Netherlands

**Keywords:** mortality salience, self-esteem, national identification, ethnocentrism, Multicultural society

## Abstract

Terror Management Theory (TMT; Greenberg et al., 1997) proposes that mortality concerns may lead people to reject other cultures than their own. Although highly relevant to multiculturalism, TMT has been rarely tested in a European multicultural society. To fill this void, two studies examined the effects of mortality salience (MS) among native Dutch people with varying levels of national identification and self-esteem. Consistent with TMT, MS led to less favorable attitudes about Muslims and multiculturalism among participants with high (rather than low) national identification and low (rather than high) self-esteem (Study 1). Likewise, MS led participants with high national identification and low self-esteem to increase their support of Sinterklaas, a traditional Dutch festivity with purported racist elements (Study 2). Together, these findings indicate that existential concerns may fuel resistance against multiculturalism, especially among people with low self-esteem who strongly identify with their nationality.

## Introduction

With modern societies becoming increasingly multicultural, growing numbers of people are regularly exposed to a diversity of cultural worldviews (Verkuyten, 2005, 2006, 2010). According to an influential social-psychological framework, Terror Management Theory (TMT; Greenberg et al., 1997), exposure to different worldviews can give rise to existential anxiety, and trigger defensive responses that are aimed at alleviating this anxiety. TMT thus seems highly relevant to the psychology of living in a multicultural society. Although TMT has been researched in hundreds of studies (for an extensive overview, see Solomon et al., 2015), the theory has been applied mostly to intergroup settings (Castano and Dechesne, 2005) international conflict (Pyszczynski et al., 2008) and multicultural issues in North America (Dupuis and Safdar, 2010; Bassett and Connelly, 2011; Motyl et al., 2011; Cohen et al., 2013). To date, however, only few studies have rarely tested the implications of TMT for living in a European multicultural society (Das et al., 2009). In the present article, we seek to fill this important gap, by examining the impact of existential concerns on multicultural attitudes among native Dutch people.

Inspired by existentialist writings (Kierkegaard, 1844/1957; Rank, 1941; Becker, 1973), TMT assumes that people's awareness of their mortality creates a potential for overwhelming terror, which is reduced by psychological defenses. One key psychological defense is formed by cultural views that offer a meaningful and orderly conception of the world. Consistent with this, many studies shown that experimentally reminding people of death leads them to defend their cultural worldviews more vigorously (for reviews, see Pyszczynski et al., 2015; Solomon et al., 2015). For instance, a frequently replicated finding is that, among American participants, mortality salience leads to greater liking of a foreign student who praises the USA and less liking for a foreign student who critiques the USA (e,g., Greenberg et al., 1990). Similar effects of mortality salience been observed in countries throughout the world, including Israel, Japan, China, the Netherlands, Spain, Australia, Iran, and others (Greenberg et al., 2008; Burke et al., 2010).

Notably, mortality salience effects tend to be more pronounced among people who strongly identify with their cultural group (Castano et al., 2002), who presumably are most invested in their culture, self-reported high identifiers who were then reminded of their mortality perceived greater homogeneity in their in-group compared to the out-group, while low identifiers showed the opposite response (Castano and Yzerbyt, 1998). Thus, existential concerns also enhance the importance of one's in-group as an unconscious defense mechanism (Dechesne et al., 2000a,b; Castano et al., 2002; Castano, 2004). Moreover, in-group identification has been shown to mediate the mortality salience effect on measures of in-group bias (Castano et al., 2002), and in a recent study this was found to be the case only when self-esteem was not enhanced (Hohman and Hogg, 2015). Thus, in-group identification may serve the secondary function of indirect self-enhancement, namely by allowing people with low self-esteem to feel significant and valuable. In support of the latter idea, one study (Arndt et al., 2002) showed that people consistently distance themselves from their in-groups when such in-group identification may undermine their self-esteem. For instance, after reading about a Hispanic drug dealer, Hispanics who in the MS condition derogated a painting attributed to a Hispanic (but not an Anglo-American) artist and distanced themselves from the Hispanic artist as much as possible.

Mortality salience effects further tend to be attenuated among people with high (rather than low) self-esteem. This is presumably because self-esteem represents a source of existential security in addition to cultural worldviews that buffers people against the notion that their life is insignificant (e.g., Harmon-Jones et al., 1997). Consistent with this, affirming people's positive conceptions of the self has been found to lower the accessibility of thoughts of death, and defensive responding following death reminders (Schmeichel and Martens, 2005).

Originally, TMT was tested in relatively mono-cultural settings in which one culture was dominant and alternative cultures were only represented by non-permanent residents (e.g., foreign students in the US) or geographically distant cultures (e.g., international conflicts). In more recent years, however, a growing number of TMT studies have begun to address how TMT is potentially relevant to genuine multicultural settings in which people encounter others on a daily basis who have cultural beliefs and practices that differ from their own. For instance, one early study by Schimel et al. (1999) showed that mortality salience led Caucasian Americans to increase the stereotyping of African Americans. recently, Bassett and Connelly (2011) observed that mortality salience increased negative reactions toward illegal aliens among Americans. Motyl et al. (2011) investigated ways to mitigate the mortality salience effect on anti-Arab prejudice among Americans. They found that reminding people of widely shared human experiences reduced the negativity toward outgroups. Finally, a study by Dupuis and Safdar (2010) showed that mortality salience led to a greater desire for assimilation of (threatening aspects of) Arab-Muslim immigrants among Canadians.

The aforementioned research was largely conducted in a North-American context. A handful of recent studies have focused specifically on European multicultural contexts. Specifically, Weise et al. (2012) found in one study that immigrants were only derogated under MS by French participants who scored high on a measure of right-wing authoritarianism. Furthermore, a German study showed that participants exposed to terrorism pictures (vs. controls) had increased prejudice against both Muslims (Study 1) and immigrants (Study 2) only when they were led to believe that literal immortality does not exist, suggesting that the effect was mediated by death-related thoughts (Kastenmüller et al., 2011). Moreover, Das et al. (2009) observed that news about terrorism increased the accessibility of death-related thoughts among native Dutch, and this effect was associated with more anti-Arab prejudice. In a similar vein, Koole et al.(2014, Study 4) found that mortality salience led native Dutch people with low self-esteem to attribute more negative emotions to Muslims.

These studies provide initial evidence that existential concerns among native Dutch may lead to more negative attitudes multicultural society in North America and in Europe. Given the importance of multicultural attitudes, we wanted to learn more about the effects of mortality salience on multicultural attitudes in Europe. In particular, we designed the present studies to investigate the potential moderating role of national identity and self-esteem in the effects of mortality salience on multicultural attitudes among native Dutch people.

In two studies, we used a subtle questionnaire to experimentally manipulate the salience of existential concerns, a procedure that was used successfully in previous TMT research among Dutch adolescents (Koole et al., 2014). To ensure the personal relevance of the multicultural attitudes that we examined, we chose to focus on societal issues that were hotly debated in Dutch society at the time of data collection (the years 2013 and 2014). Specifically, Study 1 focused on tensions between native Dutch and Muslim, mostly Moroccan and Turkish, populations within Dutch society. We manipulated mortality salience and predicted that it would subsequently increase derogation of Muslims and immigrants among native Dutch participants with high national identification and low self-esteem.

Study 2 sought to provide a conceptual replication of this pattern in the context of support for “Sinterklaas,” a traditional Dutch festivity that has been recently critiqued for containing alleged racist elements. We predicted that mortality salience would increase support for Sinterklaas among native Dutch with high national identification and low self-esteem.

## Study 1

Study 1 investigated the effects of mortality salience on attitudes toward multiculturalism among native Dutch people with varying levels national identification and self-esteem. To obtain a comprehensive test, we used five different measures of opposition to multiculturalism. First, participants indicated their feelings of warmth toward Muslims as well as three control groups: atheists, Jews and Christians. Second, participants rated to what extent a typical Dutch person and a typical Muslim would be able to experience positive and negative emotions. Third, participants evaluated an anti-Dutch essay presumably written by an international student from Morocco, a Muslim-majority country from which many people recently migrated to the Netherlands. Fourth, participants rated statements assessing their acceptance of ethnic minorities in Dutch society. Fifth and last, participants rated statements assessing their acceptance of specifically Muslim immigrants in Dutch society. Across all these measures, we expected that mortality salience would lead to more negative attitudes toward Muslims and multiculturalism, especially among native Dutch participants with high (rather than low) national identification and low (rather than high) self-esteem.

### Methods

#### Participants and design

During February-March 2013, we collected data of 144 persons. We aimed to recruit as many native Dutch students as possible in 4 weeks' time. Before any data analysis, we excluded six non-Western participants because they were not expected to identify with the Dutch people. The final dataset consisted of 138 volunteers (90 women; *M*_age_ = 20.85, *SD* = 2.93) from the Vrije Universiteit Amsterdam. Participants were randomly assigned to either a mortality salience (*n* = 72) vs. dentist salience (*n* = 66) condition. Level of national identification and self-esteem were between subjects factors and multicultural attitudes were the main dependent variable. Participants received payment or partial course credit. All participants signed an informed consent form.

#### Procedure

Participants were invited to complete a study at the psychology laboratories at the Vrije Universiteit Amsterdam. Participants were greeted by the experimenter and escorted to a private cubicle. They were informed that they were taking part in several unrelated studies that were administered together for efficiency reasons. All instructions were administered via computer. Participants were also randomly assigned to a cubicle with the light on or off. This assignment had no significant effects on our dependent variables.

First, participants filled out measures of mood, self-esteem, action-state orientation, and loneliness. We found no effects for the latter two, so they will not be discussed further. They were then randomly assigned to either the mortality salience condition or the dentist salience control condition. In the mortality salience condition, participants answered seven questions about their fear of death. In the dentist salience condition, participants answered parallel questions about their fear of visiting the dentist. They then filled out a second measure of mood.

Participants subsequently completed five worldview defense measures in a predetermined order: warmth ratings of Muslims compared to other religious groups, a measure of ethnocentric emotions, evaluations of pro-Dutch and anti-Dutch essays that were ostensibly written by Moroccan exchange students, and questionnaires measuring general acceptance of ethnic minorities in Dutch society and acceptance of Muslim immigrants in Dutch society. Participants then filled out a measure of national identification, some exploratory measures (see Appendix A for exploratory results) and the questionnaire of the condition (mortality salience vs. dentist salience) that they had not been assigned to as an extra check. Finally, participants answered some biographical questions, and were debriefed, rewarded, and thanked.

#### Materials and measures

##### Mood

Mood was measured with the brief version of the Profile of Mood States (POMS; McNair et al., 1971). Participants rated 32 emotional descriptors from 1 (not feeling the emotion at all) to 9 (strongly feeling the emotion). These descriptors were grouped into five subscales that tapped into feelings of tension, depression, vigor, anger, and fatigue. After reverse-scoring the items of the vigor subscale, all items were averaged to create a single index of negative mood for each of the two measurements (their respective Cronbach's αs were 0.85 and 0.84).

##### Self-esteem

Self-esteem was assessed with the 10-item Rosenberg (1965) self-esteem scale (RSES). Participants rated 10 items on 9-point Likert scales with higher scores representing either a negative (e.g., “All in all, I am inclined to feel that I am a failure”) or a positive view of self (e.g., “I feel that I have a number of good qualities”) on a scale of 1 (not at all) to 9 (very much). After reverse-scoring, items were averaged to create a single index (Cronbach's α = 0.89), with a higher score indicating a positive view of self.

##### Mortality salience

Participants in the mortality salience condition filled out a brief 7-item version of the Fear of Personal Death Scale (FPDS; Florian and Kravetz, 1983). Participants rated items (e.g., “I am afraid of death because I will cease to exist”) on a scale of 1 (not at all) to 5 (very much), with higher scores representing higher fear of death. This procedure has been found to reliably produce mortality salience effects (Florian and Mikulincer, 1997; Koole et al., 2014). Participants in the dentist salience condition filled out parallel items on their fear of going to the dentist (e.g., “I am afraid of the dentist because I fear losing all my teeth”).

##### Warmth ratings of muslims

To measure participants' general feelings toward Muslims, we asked participants to fill out “feeling thermometers.” The feeling thermometer has been used as a global measure of in-group and out-group feelings (e.g., Alwin, 1997; Verkuyten and De Wolf, 2002). Participants were asked to use a sliding scale to indicate their feelings of warmth toward Muslims, atheists, Jews, and Christians on a range from 0 (very cold or negative feelings) to 100 (very warm of positive feelings). Fifty degrees represented neutral feelings. Markings above 50 degrees indicated positive or warm feelings, and markings below 50 degrees indicated cold or negative feelings.

##### Ethnocentric emotions

For the ethnocentric emotions measure, we asked participants to rate whether a target person would be able to experience three negative primary emotions (fear, exhaustion, and pain), three negative secondary emotions (embarrassment, contempt, and humiliation), three positive primary emotions (affection, pleasure, and attraction), and three positive secondary emotions (admiration, hope, and surprise) on a scale of 1 (not at all) to 9 (very much) (De Dreu et al., 2011). Participants were presented with the list twice, in random order—once for a “typical” Dutch person and once for a “typical” Muslim person. Ratings were summed separately by valence for the Dutch and Muslim targets (0.55 < Cronbach's αs < 0.77). Emotion type (primary vs. secondary) did not interact with mortality salience and is not further discussed. Ethnocentrism was indicated when participants attributed (a) more positive emotions and less negative emotions to Dutch persons, and (b) less positive emotions and more negative emotions to Muslims (see also Koole et al., 2014).

##### Appreciation of anti-dutch essay

We used a well-validated measure of worldview defense (see Greenberg et al., 1992a, for an example; see Supplementary Materials for the essays we used). Participants received a pro-Dutch and an anti-Dutch essay to read and evaluate that were ostensibly written by international students from Morocco. The order of the presentation of the essays was counterbalanced. The pro-Dutch essay praised the Dutch people and their values, focusing on freedom, education and opportunities. The anti-Dutch essay was highly critical of Dutch values, focusing on the emphasis on wealth and lack of sympathy and warmth for other people. The evaluation of the essays consisted of four questions assessing participants' evaluations of the author (the extent to which participants liked the author, thought he or she used sound arguments, thought he or she was biased, and how interested they would be in meeting the author), and four questions assessing participants' evaluations of the essay (the extent to which participants agreed with the essay, felt challenged by the essay, thought the essay discussed important issues, and were negatively affected by the essay). Ratings were made on a scale of 1 (not at all) to 9 (very much). We created a separate average for the anti-Dutch essay evaluations (Cronbach's α = 0.73) and the pro-Dutch essay evaluations (Cronbach's α = 0.79). The former was our variable of interest, while the latter functioned as an extra check.

##### Acceptance of ethnic minorities in dutch society

To measure participants' acceptance of ethnic minorities within Dutch society, we used a Dutch version (Arends-Tóth and van de Vijver, 2003) of the 12-item Multicultural Ideology Scale (MIS; Berry and Kalin, 1995). Studies using the Dutch version have found clear evidence for measurement equivalence or factorial similarity of this scale across ethnic groups (Arends-Tóth and van de Vijver, 2003; Verkuyten and Brug, 2004). Sample items are “The unity of this country is weakened by Dutch of different cultural backgrounds sticking to their old ways” and “The Dutch should make more of an effort to familiarize themselves with the habits and cultural backgrounds of immigrants.” Ratings were made on scales ranging from 1 (*disagree strongly*) to 9 (*agree strongly*). After reverse-scoring, the items were averaged to create a single index (Cronbach's α = 0.84), with a higher score indicating more acceptance of ethnic minorities.

##### Acceptance of muslim immigrants in dutch society

We used four items to assess participants' acceptance of Muslim immigrants in Dutch society (e.g., “It is sensible for the Dutch government to limit the immigration of Muslims”). Ratings were made on scales ranging from 1 (disagree strongly) to 9 (agree strongly). After reverse-scoring, the items were averaged to create a single index (Cronbach's α = 0.79), with higher scores indicating more acceptance of Muslim immigrants in Dutch society.

##### National identification

We assessed importance of the Dutch identity to the self-concept by asking participants to rate three items (Verkuyten, 2005) that were similar to the items on the Identity and Membership subscales of the Collective Self-Esteem Scale (Luhtanen and Crocker, 1992). The items were: “I feel connected to the Netherlands,” “I identify with Dutch people,” and “I feel like a Dutchman.” Ratings were made on scales ranging from 1 (disagree strongly) to 9 (agree strongly). The items were averaged to create a single index (Cronbach's α = 0.84), with a higher score indicating stronger national identification.

### Results

#### Preliminary analyses

##### National identification and self-esteem

On the nine-point scale, the sample reported a mean national identification of 7.01 (*SD* = 1.42) and a mean self-esteem of 6.66 (*SD* = 1.34). National identification and self-esteem were uncorrelated, *r*_(138)_ = −0.01, *p* = 0.906.

##### Mortality salience effects on national identification

We first checked if national identification was affected by mortality salience. Past research has found that mortality salience may increase national identification (Castano et al., 2002). However, we did not expect to replicate this finding, because we measured national identification at the end of the experimental session, by which time participants presumably had already engaged their psychological defenses against mortality concerns. Nevertheless, we analyzed whether there was an effect of mortality salience on national identification, and found no significant effect, *t*_(134)_ = 0.42, *p* = 0.678. We then checked whether there was an interaction between mortality salience and self-esteem on national identification. This interaction also was not significant, *t*_(134)_ = 0.49, *p* = 0.627.

##### Mood

To check whether the experimental conditions were associated with changes in mood, we conducted a repeated-measures ANOVA with mood as a within-subject factor and mortality salience as a between-subject factor. There was no interaction effect between mortality salience and mood changes over time, *F*_(1, 136)_ = 0.21, *p* = 0.646. We also found no effect of mortality salience on mood change on any of the mood subscales, *F*_(1, 136)_ < 0.64, *p*s > 0.426. This lack of a mood effects is typically found after subtle mortality salience inductions, whose behavioral effects do not appear to be mediated by mood changes (Greenberg et al., 1997; Arndt et al., 2004).

#### Main analyses

##### Attitudes toward muslims and multiculturalism

When we separately analyzed the five attitude measures—warmth ratings of Muslims, ethnocentric emotions, appreciation of anti-Dutch essay, acceptance of ethnic minorities in Dutch society, acceptance of Muslim immigrants in Dutch society—they yielded a consistent pattern of effects of mortality salience. Furthermore, we wanted to reduce the chance for type 1 error due to alpha inflation. We therefore combined the five measures into a single index of multicultural attitudes, by first standardizing the scores and then averaging them. The reliability for this composite measure was high (Cronbach's α = 0.84). The separate results for each of the measures that we used are stated in Appendix B^1^. We coded mortality salience condition (dentist salience = −1, mortality salience = 1), standardized national identification and self-esteem scores, computed an interaction term between these variables, and simultaneously entered these factors into a multiple regression analysis predicting positive attitudes toward Muslims and multiculturalism, which was calculated by taking the mean of the standardized scores of all five measures.

The analysis yielded an effect of national identification, β = −0.37, *t*_(130)_ = −4.58, *p* < 0.001, 95% CI [−0.37, −1.14], an effect of self-esteem, β = 0.21, *t*_(130)_ = 2.68, *p* = 0.008, 95% CI [0.04, 0.26], a marginal interaction between self-esteem and mortality salience, β = 0.14, *t*_(130)_ = 1.80, *p* = 0.075, 95% CI [−0.01, 0.21], and the predicted three-way interaction between mortality salience, national identification, and self-esteem β = 0.26, *t*_(130)_ = 3.29, *p* = 0.001, 95% CI [0.07, 0.29].

**Figure 1:**
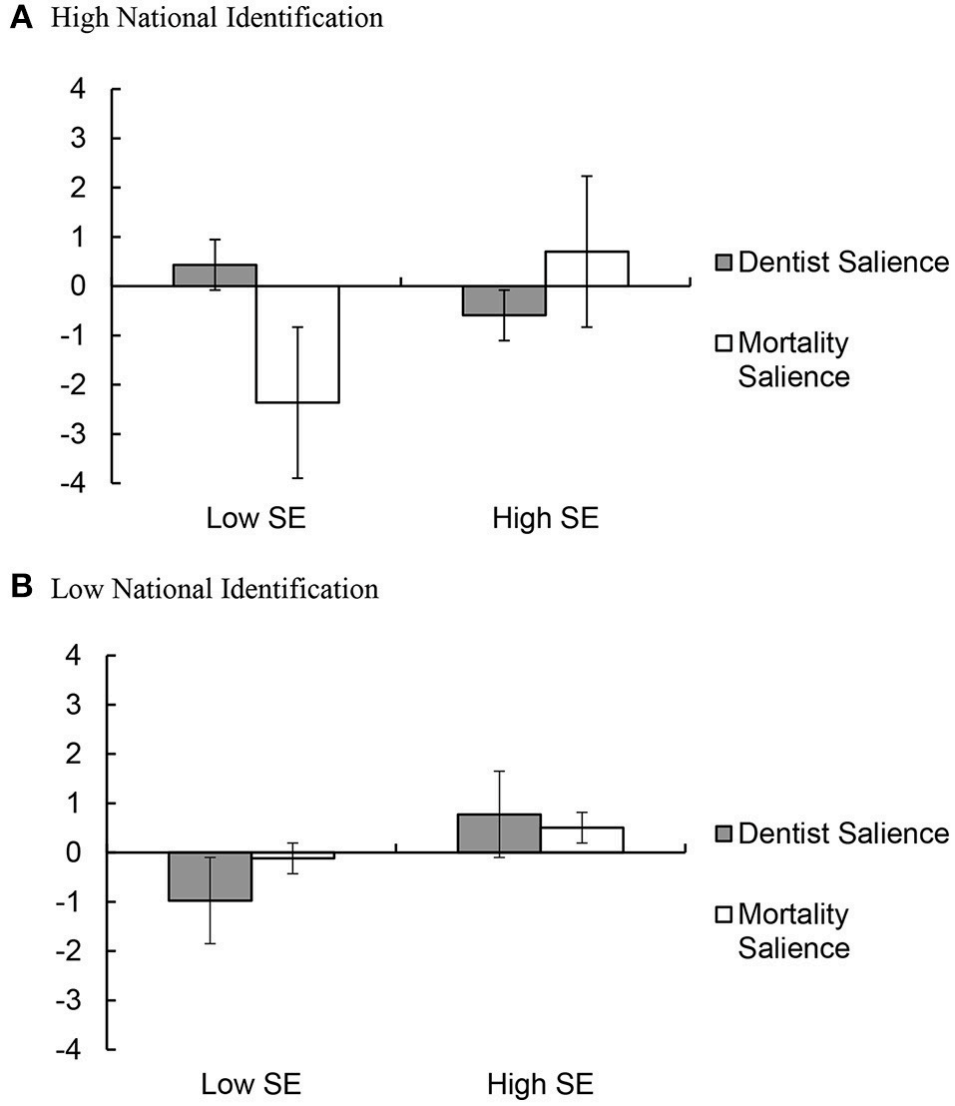
Attitudes toward Muslims and multiculturalism in Dutch society as a function of mortality salience and self-esteem among participants **(A)** high vs. **(B)** low on identification. Higher numbers indicate more positive attitudes. Low and high levels of national identification, mortality salience and self-esteem were coded as 1 *SD* below and above the mean, respectively. The overall score was calculated by taking the average standardized scores across five worldview defense measures. Standard errors are shown as error bars attached to each column.

To unpack the observed three-way interaction effect, we derived predicted means for the eight cells crossing national identification (± 1 *SD*) and self-esteem (± 1 *SD*) with mortality salience condition (Aiken and West, 1991). We first checked for effects among those who reported high vs. low national identification. As shown in Figure 1, there was no significant interaction between self-esteem and mortality salience among participants who reported low national identification, β = −0.11, *t*_(130)_ = 1.00, *p* = 0.317, 95% CI [−2.23, 0.08]. By contrast, we found a significant interaction between self-esteem and mortality salience among participants with high national identification, β = 0.38, *t*_(130)_ = 3.52, *p* < 0.001, 95% CI [0.12, 0.44].

As hypothesized, among participants with high national identification and low self-esteem, mortality salience led to less positive attitudes toward Muslims and multiculturalism, β = −0.37, *t*_(130)_ = −2.23, *p* = 0.023, 95% CI [−0.51, −0.04]. In addition, and unexpectedly, mortality salience also had a significant impact among participants with high national identification and high self-esteem, β = 0.39, *t*_(130)_ = 2.66, *p* = 0.009, 95% CI [0.07, 0.50], such that mortality salience increased positive attitudes toward Muslims and multiculturalism among the latter group.

### Discussion

As expected, mortality salience interacted with national identification and self-esteem to predict attitudes toward Muslims and multiculturalism. Among participants with low national identification, mortality salience had no effect. The observed lack of mortality salience effects among participants with low national identification suggests that low national identification should not be equated with positive attitudes toward multiculturalism. Specifically, many people who do not identify with their nationality may still be skeptical about multiculturalism, perhaps because they are opposed to all forms of nationalism (even from other cultures) or because they feel indifferent toward various cultural groups (including their own). For these reasons, multiculturalism may not have an anxiety-buffering function for people with low national identification. People with low national identification may hence remain relatively lukewarm about multiculturalism, even after they have been reminded of their mortality (Greenberg et al., 1997).

By contrast, among participants with high national identification, mortality salience interacted with level of self-esteem. The pattern for participants with low self-esteem was as predicted by TMT: Among low self-esteem participants, mortality salience decreased positive multicultural attitudes. Overall, these findings confirm the relevance of existential concerns for understanding multicultural attitudes, while suggesting that existential concerns are especially relevant for people high on national identification and low on self-esteem.

Unexpectedly, mortality salience also influenced multicultural attitudes among participants high on national identification and high on self-esteem. In the dentist salience condition, high self-esteem participants reported less positive multicultural attitudes than in the mortality salience condition. Because the effects of mortality salience among people with high self-esteem were not predicted a priori, we are reluctant to attach much theoretical significance to them. Still, one possibility is that people high on national identification with high self-esteem turn to their sense of self-worth as a personal anxiety buffer, which leads them to relinquish some of their national identification when existential concerns become salient. Pending replication, however, this interpretation must remain speculative.

## Study 2

In Study 2, we sought to conceptually replicate and extend our findings in Study 1. Instead of Dutch-Muslim relations, we shifted our focus to the relations between Dutch majority and Black minority members. At present, the Netherlands has a sizable ethnic Black subpopulation, many of which originate from its former colonies of Surinam and the Dutch Antilles. We investigated an issue that is highly meaningful in the contemporary Dutch-Black relations, namely the contested Dutch tradition of Sinterklaas. this issue provided a direct analog to the attitudes toward Muslims that were measured in Study 1. The celebration of “Sinterklaas” is held annually between November and December. This traditional Dutch children's festivity includes the folklore character called “Zwarte Piet” (“Black Pete”), who appears with blackface, afro hair, red lips, and golden hoop rings. This appearance has led some to conclude that Zwarte Piet must be a racist figure. Indeed, a recent United Nations committee that investigated Zwarte Piet called it “a return to slavery” (Bloem, 2013). Furthermore, in July 2014, a Dutch judicial ruling announced that Zwarte Piet was an offensive stereotype. In the wake of this ruling and the surrounding publicity, the traditional celebration of Sinterklaas has become increasingly protested. In the year of the study, for instance, 90 protesters were arrested during the ceremonial arrival of Sinterklaas (Iyengar, 2014).

The recent opposition to Zwarte Piet can be viewed as an attempt to end (or, at least, substantially alter) a quintessential Dutch tradition, and thus, symbolically, as an existential threat to Dutch culture. We therefore predicted that attitudes toward Zwarte Piet would display the same kind of interaction between mortality salience, national identification, and self-esteem, as in Study 1. Specifically, we predicted that mortality salience would lead to more positive attitudes toward Zwarte Piet among our native Dutch participants, especially those high on national identification and low on self-esteem. This part of Study 2 could thus be regarded as a conceptual replication of Study 1.

### Methods

#### Participants and design

Our aim was to collect as many participants as possible in the weeks from September 22nd until the end of November. We collected data of 213 persons. Prior to data analysis, we excluded 39 people who reported having a non-Western ethnic background because they were not expected to display Dutch identification. The final dataset thus consisted of 174 volunteers (139 women; *M*_age_ = 19.87, *SD* = 3.39) from the Vrije Universiteit Amsterdam. They were randomly assigned to either the high (*n* = 83) or low (*n* = 91) mortality salience condition. As in Study 1, national identification and self-esteem were between-subjects factors. The main dependent variable was the endorsement of the traditional celebration of the popular but controversial Sinterklaas. Participants received payment or partial course credit. All participants signed an informed consent form.

#### Procedure, materials and measures

Our measures of pre-manipulation mood (Cronbach's α = 0.84), self-esteem (Cronbach's α = 0.91), national identification (Cronbach's α = 0.89), and mortality salience manipulation were the same as in Study 1. We again measured mood for a second time after the threat manipulation (Cronbach's α = 0.82). This time, however, we also included two additional mood items, namely “afraid” and “startled,” following recent research showing that mortality salience manipulations increase negative affect especially on fear or terror related items (Lambert et al., 2014).

##### Support for the sinterklaas tradition

Participant then filled out an 8-item questionnaire measuring endorsement of the traditional celebration of Sinterklaas (e.g., “Zwarte Piet should remain a part of the Sinterklaas tradition,” “Celebrating Sinterklaas is a part of the Netherlands”) on a scale of 1 (*do not agree at all*) to 7 (*completely agree*). Finally, participants filled out a measure of national identification (Cronbach's α = 0.89) and exploratory measures of appreciation of Dutch caricatures, self-stereotyping, Theory of Mind and Social Dominance Orientation. The results of all exploratory measures are described in Appendix A.

### Results

#### Preliminary analyses

##### National identification and self-esteem

On the seven-point scale, participants of Study 2 reported a mean national identification of 5.38 (*SD* = 1.28) and a mean self-esteem of 5.06 (*SD* = 1.07).The two measures were uncorrelated, *r*_(174)_ = 0.07, *p* = 0.372. We also performed a one-way ANOVA to ensure that the experimental conditions did not differ in national identification, and indeed found no effect, *F* < 1.

##### Mood

To check whether the experimental conditions and levels of self-esteem were associated with changes in mood, we conducted a repeated-measures ANOVA with mood as within-subject factor, mortality salience as between-subject factor, self-esteem as covariate, and two interaction terms, one between mortality salience and mood, and one between self-esteem and mood. There was a main effect of time *F*_(1, 171)_ = 11.09, *p* = 0.001, $\eta_{\text{p}}^{2}$ = 0.06, and interaction effects between change in mood and mortality salience, *F*_(1, 171)_ = 16.83, *p* < 0.001, $\eta_{\text{p}}^{2}$ = 0.09, and between change in mood and self-esteem. Thus, there was a greater increase in negative mood in the mortality salience condition from Time 1 (*M* = 2.96, *SD* = 0.71) to Time 2 (*M* = 3.10, *SD* = 0.84) compared to the change in the dentist salience condition from Time 1 (*M* = 3.07, *SD* = 0.98) to Time 2 (*M* = 2.99, *SD* = 0.99) and self-esteem was associated with a stronger decrease in negative mood over time. We found no effects with the “afraid” and “startled” items.

Because of the difference in mood between the mortality salience and the dentist salience condition, we repeated the analyses reported below using changes in negative mood and its interaction with condition as a covariate. These analyses did not yield any different results compared to the analyses without the added covariates. Thus, consistent with prior TMT research (Greenberg et al., 1997; Lambert et al., 2014), mood changes did not explain our findings.

#### Main analyses

##### Support for the sinterklaas tradition

**Figure 2:**
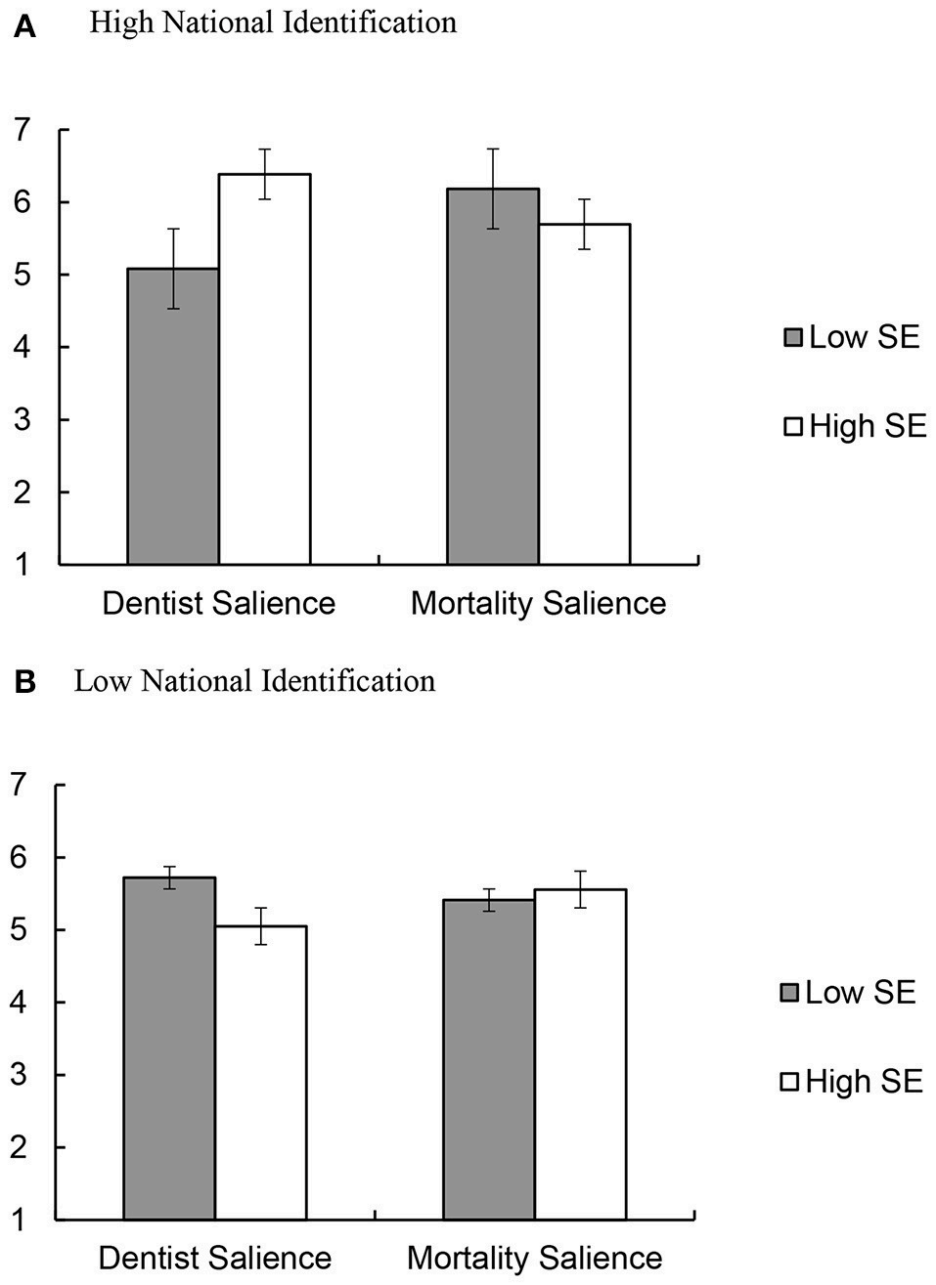
Support for the Sinterklaas tradition as a function of mortality salience and self-esteem among participants **(A)** high vs. **(B)** low on identification. Low and high levels of identification, mortality salience and self-esteem were coded as 1 *SD* below and above the mean, respectively. The items were rated on a scale from 1, *completely disagree* to 7, *completely agree*.

To test our prediction that mortality salience would increase support for Sinterklaas among native Dutch with high national identification and low self-esteem, we conducted a multiple regression analysis. This yielded a main effect of national identification, β = 0.23, *t*_(166)_ = 3.02, *p* = 0.003, 95% CI [0.08, 0.38], and a significant three-way interaction between mortality salience, national identification, and self-esteem, β = −0.15, *t*_(166)_ = −2.00, *p* = 0.047, 95% CI [−0.31, −0.002]. This interaction effect is visually displayed in Figure 2.

To interpret the interaction, we derived predicted means for the eight cells crossing self-esteem (± 1 *SD*) and identification (± 1 *SD*) with mortality salience condition (Aiken and West, 1991). We then performed simple slope analyses for participants high and low on national identification. As expected, there were no effects among participants low on national identification, *t*s < 0.92, *p*s > 0.361. Also as expected, among participants high on national identification, we found a marginally significant interaction between self-esteem and mortality salience, β = −0.21, *t*_(166)_ = −1.91, *p* = 0.057, 95% CI [−0.43, 0.01]. Simple-slopes analyses showed that, in the dentist salience condition, self-esteem was associated with more support for the Sinterklaas tradition among participants high on national identification, β = *0*.30, *t*_(166)_ = 1.99, *p* = 0.048, 95% CI [0.003, 0.61]. However, in the mortality salience condition, people with low self-esteem to become equally supportive of the Dutch tradition as people with high self-esteem, β = −0.12, *t*_(166)_ = −1.73, *p* = 0.466, 95% CI [−0.43, 0.20].

### Discussion

The results of Study 2 showed a pattern that was theoretically similar to the one observed in Study 1: mortality salience led people high on national identification and low self-esteem to show more support for the traditional, but contested Dutch celebration of Sinterklaas. This pattern fits with the notion that existential concerns fuels resistance against multiculturalism, primarily among people who strongly identify with their nationality and who have low self-esteem. Furthermore is important to note that support for Sinterklaas is generally high for reasons unassociated with defensiveness. Indeed, it is a uniquely Dutch tradition loved by many Dutch, Thus, it is not surprising to find the highest amount of support among people with high self-esteem in the control condition. However, increased support for Sinterklaas as a response to MS suggests that support for the tradition can also be motivated by worldview defense.

## General discussion

Though modern societies are becoming increasingly multicultural, multiculturalism continues to meet fierce opposition. The results of the present two studies suggest that at least some of the opposition to multiculturalism may be fueled by existential concerns. In Study 1, native Dutch who report high national identification and low self-esteem showed defensive derogation of immigrants and became less supportive of multiculturalism in response to heightened mortality salience. We found a similar effect pattern in Study 2, where mortality salience led native Dutch with low self-esteem and high national identification to become more supportive of a Dutch traditional feast that was contested by minority groups. Thus, native Dutch with high national identification and low self-esteem may use their nationality an as indirect way to gain a sense of esteem and significance (e.g., self-enhancement). These findings fit well with research showing that people may deal with existential concerns by joining and defending cultural groups that allow them to indirectly enhance their sense of self-worth and significance (see also Dechesne et al., 2000a; Arndt et al., 2002; Castano and Dechesne, 2005).

At the same time, our findings suggest that opposition to multiculturalism is not a universal response to mortality salience. First, mortality salience did not increase negative multicultural attitudes among people high in self-esteem in both of the present studies. Indeed, high self-esteem people even became more positive in their multicultural attitudes in Study 1. Although the latter effect was not replicated in Study 2, the overall pattern that we observed suggests that a sense of positive self-worth may provide substantial psychological protection against the existential anxiety that may arise from multicultural exchanges. Notably, other terror management research has shown that the anxiety-buffering effects of self-esteem do not only arise from dispositional self-esteem, but also from momentary boosts in state self-esteem (Greenberg et al., 1992b). Future research may examine if such momentary increases in self-esteem may also help to lower opposition to multiculturalism, especially under conditions of mortality salience.

The present research inevitably has limitations. First, even though the present studies were conducted in a behavioral laboratory, they focused on highly debated societal issues in a specific timeframe. This enhanced societal meaningfulness, but it also means that it may be difficult for future studies on the same or a similar topic to replicate this research. We also could not control changes in perceived and realistic threat caused by salient events in the Netherlands, such as the continuing increase in resistance to the Black Pete character as we conducted our studies^2^. Second, our measures of national identification were administered at the end of the experimental session. This gives rise to the possibility that the measures were influenced in some way by mortality salience. Although our manipulation checks found no evidence for such an influence, it remains desirable to vary the timing of national identification measures in future research. Last, it should be acknowledged that the present studies had relatively small sample sizes. To obtain conclusive evidence for our observed three-way interaction effects, it will be necessary to conduct studies using substantially larger participant samples to find a medium effect size (see Wuensch, 2015, March 18).

In conclusion, the present research found that concerns about one's mortality and existential significance may foster resistance to multiculturalism. Given the universality of existential concerns, some amount of friction may be inevitable in multicultural societies. At the same time, the present findings provide some room for optimism. After all, our findings show that only about a quarter of our samples increased their opposition to multiculturalism under mortality salience, namely people with high national identification and low self-esteem. The overwhelming majority of our participants thus showed little or no increase in opposition to multiculturalism under heightened mortality salience. For achieving a less defensive stance to multiculturalism, a positive sense of self-worth appears to be the most vital psychological resource. Treating people with dignity and respect may thus create the optimal conditions for meeting the challenges of living in a multicultural society.

## Ethics statement

This study was carried out in accordance with the recommendations of the VCWE ethical committee of the Vrije Universiteit Amsterdam. All subjects gave written informed consent in accordance with the Declaration of Helsinki. The protocol was approved by the VCWE committee.

## Author contributions

MT came up with the study concept; MT and SK designed the studies, analyzed the data, and wrote the manuscript. All authors approved the final version of the manuscript before submission.

### Conflict of interest statement

The authors declare that the research was conducted in the absence of any commercial or financial relationships that could be construed as a potential conflict of interest.

## Footnotes

^1^Briefly, we found the expected three-way interactions for the warmth ratings of Muslims, the evaluation of the anti-Dutch essay, and the acceptance of Muslims in Dutch society. A, although there were minor differences in the patterns of means, the general pattern of the interactions was similar across the different dependent measures.

^2^See Appendix E for an attempt to replicate some of our findings among an online student sample as well as a short discussion on the impact of unexpected societal events on the Dutch multicultural context.
