# Supplementary material for: Terror Management in a Multicultural Society: Effects of Mortality Salience on Attitudes to Multiculturalism Are Moderated by National Identification and Self-Esteem Among Native Dutch People
Source: Front Psychol. 2018 May 15;9:721. doi: 10.3389/fpsyg.2018.00721 (PMC5962795; doi:10.3389/fpsyg.2018.00721)
Supplement: Supplementary file 1 [file Presentation_1.PDF]

Exact Dutch instructions used in our Study (and English translations)

Study 1

Starting screen
Welkom! Dit onderzoek bestaat uit twee onderdelen met losse vragenlijsten over uiteenlopende onderwerpen. Voor de duidelijkheid: al je gegevens worden vertrouwelijk en anoniem behandeld en zijn alleen bestemd voor wetenschappelijke doeleinden. Mocht je vragen hebben of er iets onduidelijk zijn, spreek dan de proefleider aan. Denk niet te lang over je antwoorden na. Het gaat er om dat je op je gevoel afgaat. Zet je mobiel uit voordat je verder gaat.
Welcome! This research consists of twee parts of unrelated questionnaires about various topics. To be clear: all your data will be treated confidentially and anonymously, and are only used for scientific purposes. If you have questions or if anything is unclear to you, please address the experimenter. Do not think too long about your answers. We are interested in your gut feeling. Turn off your mobile phone before you continue.

POMS instructions
Je krijgt zo dadelijk een aantal vragen omtrent je stemming op dit moment. Toets telkens het cijfer in dat het beste bij je huidige stemming past.
You will now be presented with a number of questions about your mood. Enter the number that best represents your current mood.

Questionnaire instructions
Je krijgt nu een aantal stellingen. Geef voor iedere stelling aan in hoeverre deze op jou van toepassing is. Dat was het eerst in je op komt is het beste antwoord.
You will now be presented with a number of statements. Rate each statement on whether it applies to you. Your first reaction is the best answer.

Mortality Salience Manipulation<br>*Mortality Salience Manipulation (statements were presented on the screen one at a time)*
I'm afraid of death, because then I will have to part with life.

I'm afraid of death, because then I permanently will cease to exist.<br>I'm afraid of death, because I fear the disease and pain that mostly accompanies dying.<br>I'm afraid of death, because it is so final.<br>I'm afraid of death, because my body will decay.<br>I'm afraid of death, because I expect that the people I leave behind will quickly forget about me.<br>I'm afraid of death, because I expect that everything will just continue as usual after my death.
Ik ben bang voor de dood, want dan moet ik afscheid nemen van het leven.<br>Ik ben bang voor de dood, want dan hou ik voorgoed op te bestaan.<br>Ik ben bang voor de dood, want ik zie op tegen de ziekte en pijn waarmee sterven meestal gepaard gaat.<br>Ik ben bang voor de dood, want het is zo definitief.<br>Ik ben bang voor de dood, want dan vergaat mijn lichaam.<br>Ik ben bang voor de dood, omdat ik verwacht dat mijn nabestaanden mij snel zullen vergeten.<br>Ik ben bang voor de dood, omdat ik verwacht dat alles na mijn dood gewoon zal doorgaan alsof er niets aan de hand is.

Fear of dentist visit (Control Manipulation)
Ik ben bang voor de tandarts, want dan moet ik mijzelf overleveren aan iemand anders.<br>Ik ben bang voor de tandarts, want ik ben bang dat ik uiteindelijk al mijn tanden kwijtraak.Ik ben bang voor de tandarts, want ik zie op tegen de pijn waarmee een tandartsbezoek meestal gepaard gaat.<br>Ik ben bang voor de tandarts, want ik heb mijn tanden niet altijd goed gepoetst.<br>Ik ben bang voor de tandarts, want het geluid van de boor gaat door merg en been.<br>Ik ben bang voor de tandarts, omdat ik verwacht dat een bezoek aan de tandarts mij veel geld gaat kosten.<br>Ik ben bang voor de tandarts, omdat ik bang ben dat ik veel gaatjes heb.
I'm afraid of the dentist, because then I have to surrender myself to someone else.<br>I'm afraid of the dentist, because I'm afraid that I will eventually lose all my teeth.<br>I'm afraid of the dentist, because I dread the pain that usually accompanies a visit to the dentist.

I'm afraid of the dentist, because I have not always brushed my teeth very well.<br>I'm afraid of the dentist, because the sound of the drill penetrates my whole body.<br>I'm afraid of the dentist, because I expect that a visit to the dentist will cost me dearly.<br>I'm afraid of the dentist, because I'm afraid I will have many cavities.

Feeling thermometer instructions
Bij deze opdracht moet u de 'GEVOELSTHERMOMETER' gebruiken om aan te geven hoe u over verschillende groepen denkt. U mag iedere temperatuur tussen 0 en 100 graden gebruiken, maar u moet wel één getal kiezen. 100 graden betekent zeer warme of positieve gevoelens en 0 graden betekent zeer koude of negatieve gevoelens.
For this task you must use the FEELING THERMOMETER to indicate how you think of certain groups. You may use any temperature between 0 and 100 degrees, but you must choose a single number. 100 degrees indicates very warm or positive feelings and 0 degrees indicates very cold or negative feelings.

Feeling thermometer task

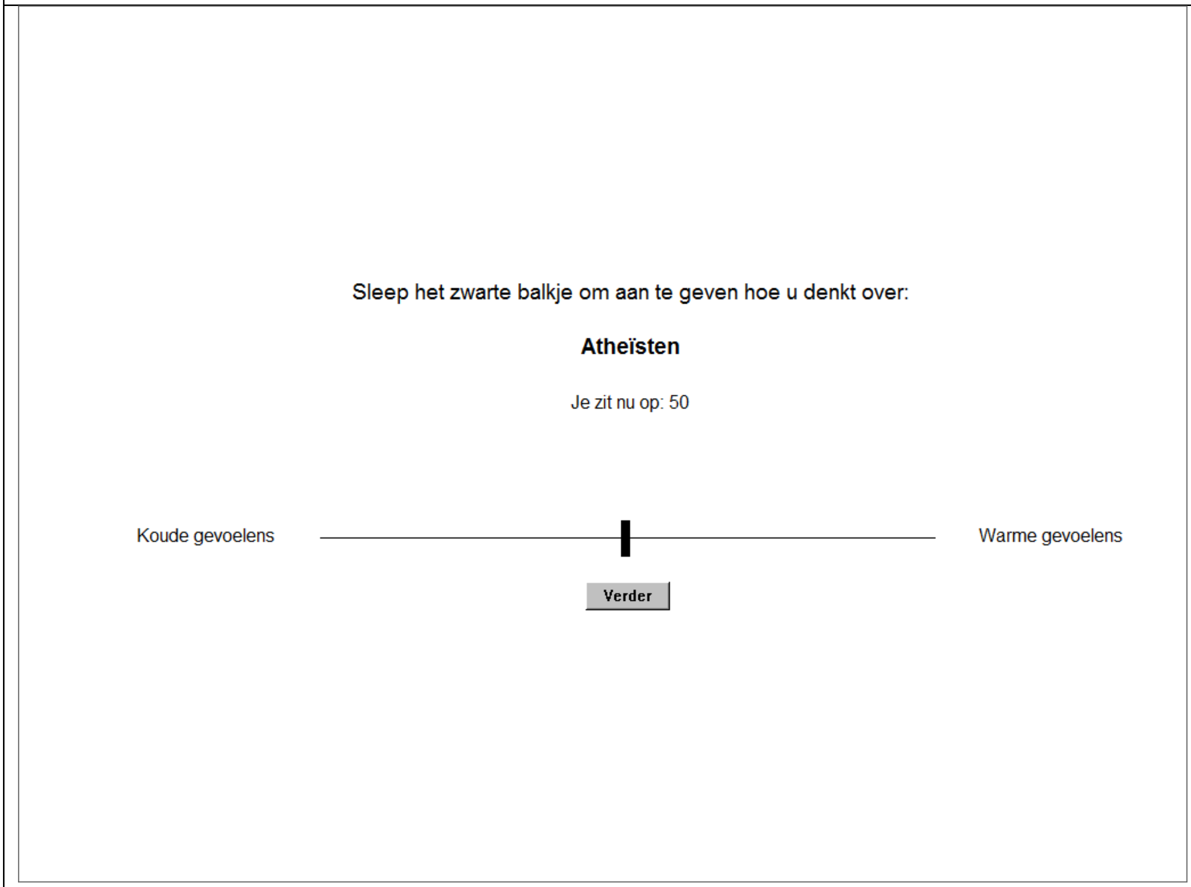

Drag the black beam to indicate how you think of: Atheists. Cold feelings – warm feelings.

Ethnocentric emotions
"Uit eerder onderzoek blijkt dat mensen op een hele intuïtieve manier kunnen begrijpen welke emoties door anderen wordt ervaren. Mensen zijn dus ook in staat om subtiele verschillen in emotiebeleving van verschillende groepen mensen op te merken. Bij deze taak vragen wij je om de emotiebeleving van enkele groepen te beoordelen."
**“**Previous research has shown that people can understand intuitively which emotions are experiences by others. People are thus able to notice subtle differences in emotional experience. For this task we ask you to judge the emotional experience of several groups.”

Essay task
“Hierna volgen twee verschillende teksten. Deze teksten zijn afkomstig uit een eerder onderzoek waarbij we studenten van allochtone afkomst naar hun mening over Nederland hebben gevraagd. Lees de teksten zorgvuldig en vul de vragenlijsten in die erop volgen. Denk niet te lang over je antwoorden. Maak bij je antwoorden gebruik van je eerste, intuïtieve oordeel.”
“You will read two different texts. They were taken from a previous study in which students of ethnic minority students were asked about their opinions of the Netherlands. Read the texts carefully and answer the questionnaires that follow. Don’t think too long about your answers. Act on your first impression.”

Pro-Dutch essay
"Het eerste dat mij opviel toen ik in Nederland kwam, was de vrijheid die mensen hier hebben. Je kunt hier alles doen wat je wilt, en je kunt hier ook een goede opleiding krijgen. Iedereen kan in dit land bereiken wat hij wil, en voor mensen waar het slecht mee gaat zijn er goede sociale voorzieningen. Natuurlijk zijn er ook slechtere kanten aan Nederland, maar die heeft ieder land. En als je in Nederland iets aan je problemen wil doen, dan kan dat en willen mensen helpen. In Marokko moeten wij vechten om iets gedaan te krijgen, maar hier kan over alles gepraat worden. Nederland is een echte democratie. Ik ben blij dat ik hier kon komen en om meer te leren over het leven in Nederland."
**“**When I arrived in Netherlands, the first thing I noticed was the total freedom that people

have here. There are good opportunities for everyone, and the education here is excellent. You can achieve what you want here. There is a good system that protect people who have few chances. Of course there are weak points in Netherlands, but that is true of any country. If you want to fix problems in the Netherlands, people want to help. In Morocco we have to fight to get something done, but here you can discuss everything. The Nederlands is a true democracy. I am happy that I could come to the Netherlands and learn about life here.”

Anti-Dutch essay
"Toen ik Marokko verliet en naar Nederland ging, dacht ik dat Nederland een land was waar je echt vrij kunt zijn en tolerantie echt belangrijk is. Ik kwam er snel achter dat de Nederlandse vrijheid te ver gaat. Volgens mij kunnen Nederlanders die vrijheid helemaal niet aan. Drugs, sex en geweld. Alles kan hier, en niemand durft meer te corrigeren. Het enige wat Nederlanders interesseert is geld en snelle bevrediging. Echte sympathie en warmte voor elkaar kom je hier niet tegen. Ik vind het vreemd dat Nederlanders zoveel kritiek hebben op andere landen. Ze zouden ook naar hun eigen land mogen kijken. Ik ben blij dat ik hier weg kan gaan, want ik geloof niet dat iemand hier gelukkig kan worden."
"When I left Morocco and came to the Netherlands, I thought that the Netherlands was a country where you can be free and tolerance is important. But I realized that the freedom here goes too far. I do not think that the people from the Netherlands can handle it. Drugs, sex and violence. Everything is possible here and no one can object. Dutch people only care about money and quick satisfaction. You will not find true sympathy and warmth here. I find it strange that Dutch people criticize other countries so much. They have many problems of their own. I am glad to leave this place. I do not think anybody can be happy here.”

Appreciation of anti-Dutch essay
In welke mate vind je dat de schrijver van de tekst goede argumenten gebruikt?<br>In welke mate vind je dat de schrijver van de tekst vooroordelen heeft?<br>Wat vind je van de tekst?<br>Wat vind je van de schrijver van de tekst?<br>Wil je de schrijver van de tekst ontmoeten?<br>In hoeverre voel je je aangesproken door de tekst?

Vind je dat in de tekst belangrijke zaken worden besproken?<br>Voel je je positief of negatief aangesproken door de tekst
In welke mate vind je dat de schrijver van de tekst goede argumenten gebruikt?<br>In welke mate vind je dat de schrijver van de tekst vooroordelen heeft?<br>Wat vind je van de tekst?<br>Wat vind je van de schrijver van de tekst?<br>Wil je de schrijver van de tekst ontmoeten?<br>In hoeverre voel je je aangesproken door de tekst?<br>Vind je dat in de tekst belangrijke zaken worden besproken?<br>Voel je je positief of negatief aangesproken door de tekst

Attitudes toward multiculturalism/Muslims in Dutch society
Geef nu aan in hoeverre je het eens bent met de volgende stellingen op een schaal van: 1 (helemaal wel) tot 9 (helemaal niet)
Indicate whether you agree with the following statements on a scale of: 1 (completely agree) to 9 (completely disagree)

Study 2

Starting screen
Welkom bij dit onderzoek!<br><br>Dit onderzoek bestaat uit enkele korte onderzoeken over internethumor en de Nederlandse samenleving. Deze onderzoeken worden samen afgenomen om tijd te besparen.<br><br>Het invullen van de vragenlijsten duurt ongeveer 10 minuten en je krijgt hier 20 credits voor. Als je besluit mee te doen, ga je akkoord met het volgende:<br><br>- Het is de bedoeling dat je de vragenlijsten **in je eentje** invult. Zorg ervoor dat niemand mee kijkt. Zorg er ook voor dat je zo min mogelijk wordt onderbroken tijdens dit

onderzoek. Als je je in een onrustige omgeving bevindt, dan hebben we liever dat je deze vragenlijst een andere keer invult.
- Het is erg belangrijk dat je de inhoud van dit onderzoek de komende weken NIET deelt met je studiegenoten. Hiermee kun je de resultaten namelijk beïnvloeden.
- We vragen je straks om je VU-net-ID als je mee doet voor credits. Direct na afname van het onderzoek wordt jouw VU-net-ID ontkoppeld van jouw onderzoeksdata. Jouw anonimiteit blijft dus gegarandeerd.
- Al je gegevens worden vertrouwelijk en anoniem behandeld en zijn alleen bestemd voor wetenschappelijke doeleinden.
- Jouw deelname aan dit onderzoek is vrijwillig. Je mag je deelname op elk moment eindigen door het venster te sluiten. Je kunt dan niet opnieuw meedoen.

Als je met de bovenstaande voorwaarden akkoord gaat, kun je met het onderzoek beginnen door naar de volgende pagina te gaan.

Welcome to this research!

This research consists of several short (pilot)studies about personality, political preference and internet humor. The studies are administered together to save time.

Answering the questions takes 10 minutes and you will receive 20 credits. If you decide to continue, you agree to the following:

- You are expected to fill out the questionnaires **on your own**. Make sure that no one is watching over your shoulder. Make sure interruptions are kept to a minimum. If you are in a noisy environment, we prefer that you do this another time.
- It is very important that you DO NOT share the contents of the research with any of your study mates. This may impact the results.
- To award you the credits, we ask for your VU-net-ID. We will decouple this information from your personal info immediately after finishing with thie research.- Your anonymity will therefore still be guaranteed.
  All your data will be treated confidentially and anonymously and will solely be used for scientific purposes.

- Your participation is completely voluntary. You may quit this study at any time by

closing the browser window. You may not attempt participation again.<br>If you agree to the terms described above, you may start with the research by continuing to the next page.

Evaluation of caricatures
"Be Like"-pagina’s zijn momenteel een grote hit op Facebook. Op "Be Like"-facebookpagina’s verschijnen foto’s en korte teksten over vooroordelen of gebruiken van een bepaalde bevolkingsgroep. "Be Like" betekent "doen als" of "doen zo".<br><br>“Tattas Be Like” is een pagina die de gewoonten van Nederlanders op de hak neemt. “Tatta” betekent namelijk “aardappel” en verwijst naar het idee dat Nederlanders echte aardappeleters zijn. "Tattas Be Like" betekent dus "Nederlanders doen zo".<br><br>Wij willen deze afbeeldingen gebruiken voor een onderzoek naar verschillende soorten internethumor die momenteel populair zijn. Zo meteen krijg je steeds een afbeelding te zien afkomstig van de “Tattas Be Like”-pagina.<br><br>Het is de bedoeling dat je aangeeft hoe grappig je elke afbeelding vindt.<br><br>Je kunt direct beginnen.
“Be like”-pages are a big hit on Facebook. On “Be Like”-Facebook pages photos and short statements are presented about stereotypes or customs of a specific societal group. “Be Like” means “act like” or “Do this”.<br><br>“Tattas Be Like” is a page that makes fun of Dutch customs. “Tatta means “potato” and refers to the Dutch who are frequent consumers of potatoes. “Tattas Be Like” therefore means Dutch people are like this”.<br><br>We want to use the images for a study on different types of internet humor that are popular at the moment. You will be shown an image from the “Tattas Be Like”-page.<br><br>You are asked to rate how funny you find the images.<br><br>You may start at any time.

Caricatures of the Dutch with explanations
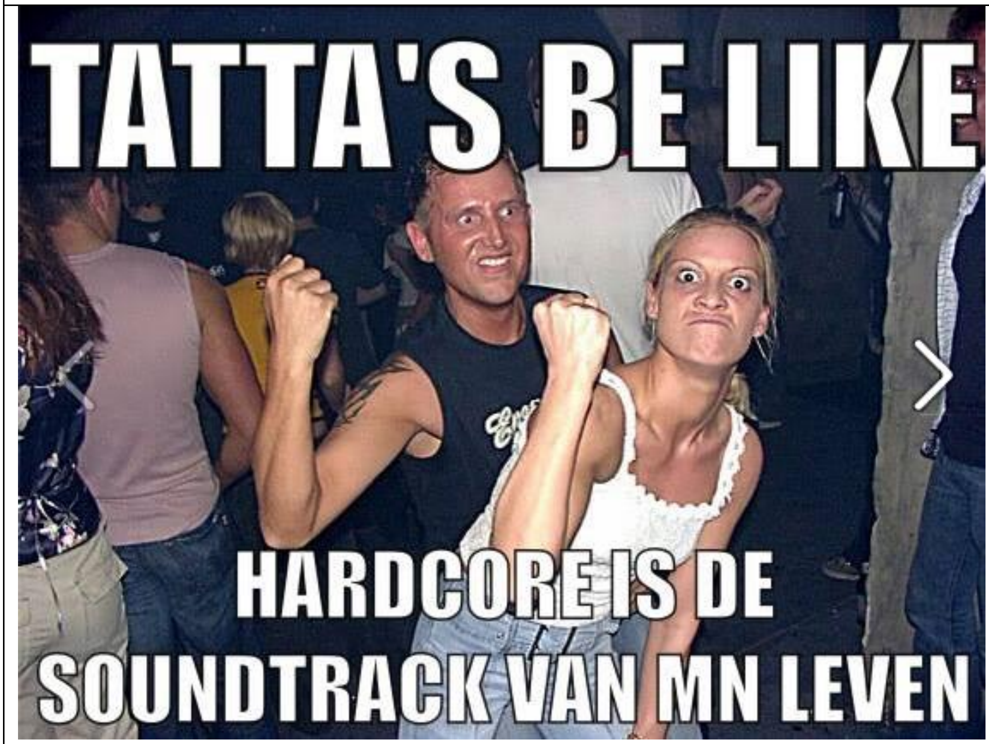
Tatta's be like: **Hardcore is the soundtrack of my life/existence**.<br>Refers to a type of music, Hardcore, that a lot of Dutch youth are (really) into.
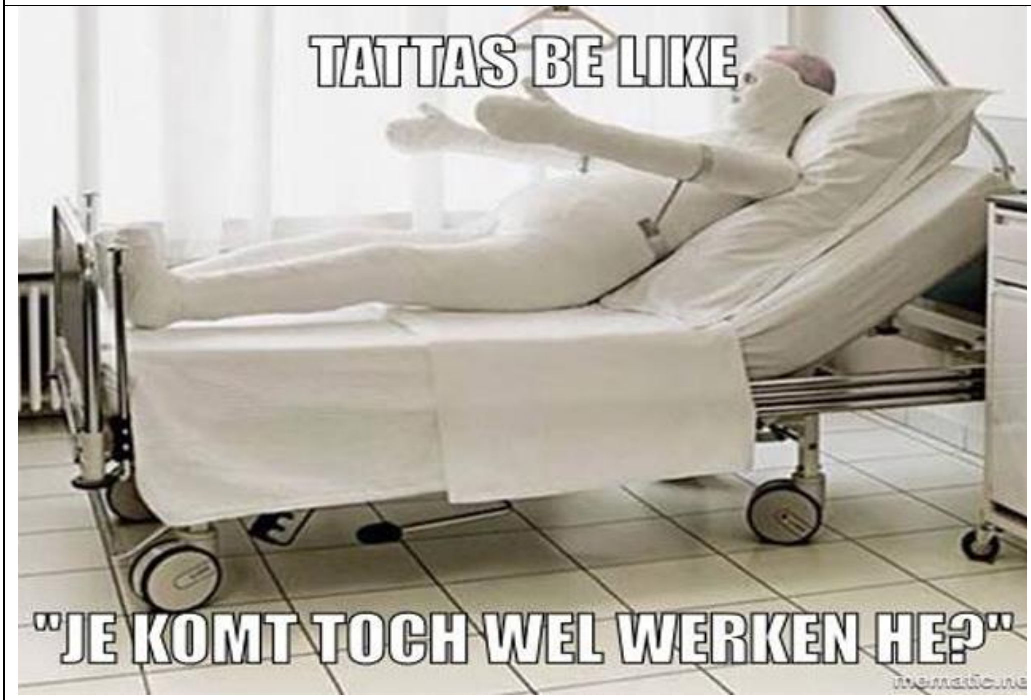
Tatta's be like: **"You are still coming to work, right?"**<br>Refers to Dutch peoples' overly serious, a no-nonsense work-ethic and how they expect the same of others.

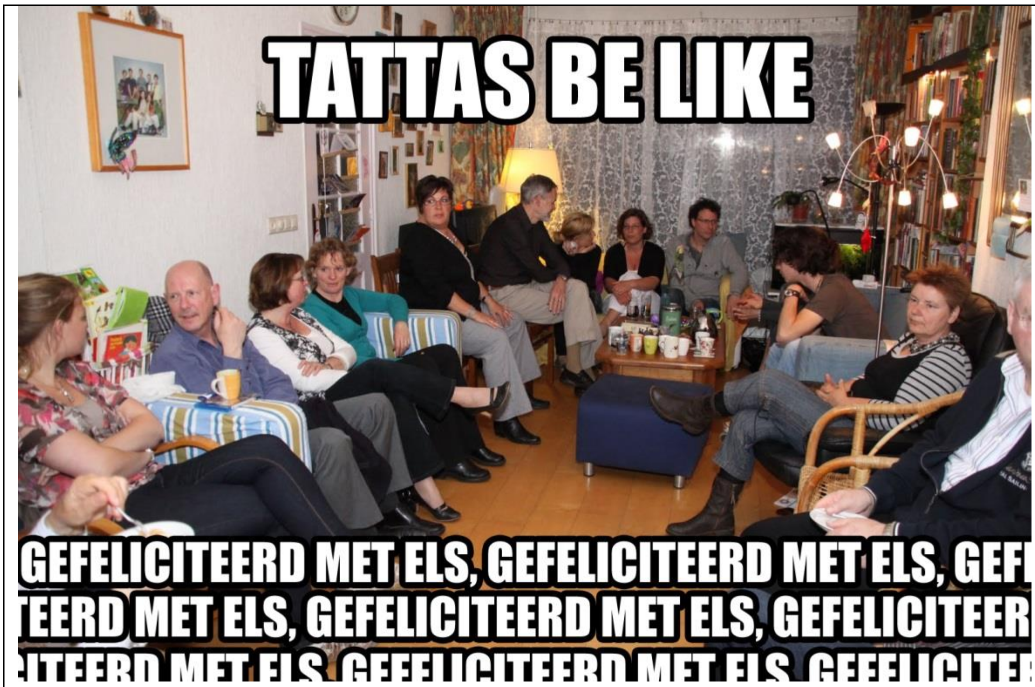

Tatta’s be like: **Congratulations with Els (a typical Dutch name)**

Refers to a very typical Dutch way to celebrate a birthday, namely in the (crowded) living room. Each person is typically congratulated with the birthday of the person’s who’s celebrating their birthday. This is a Dutch custom.

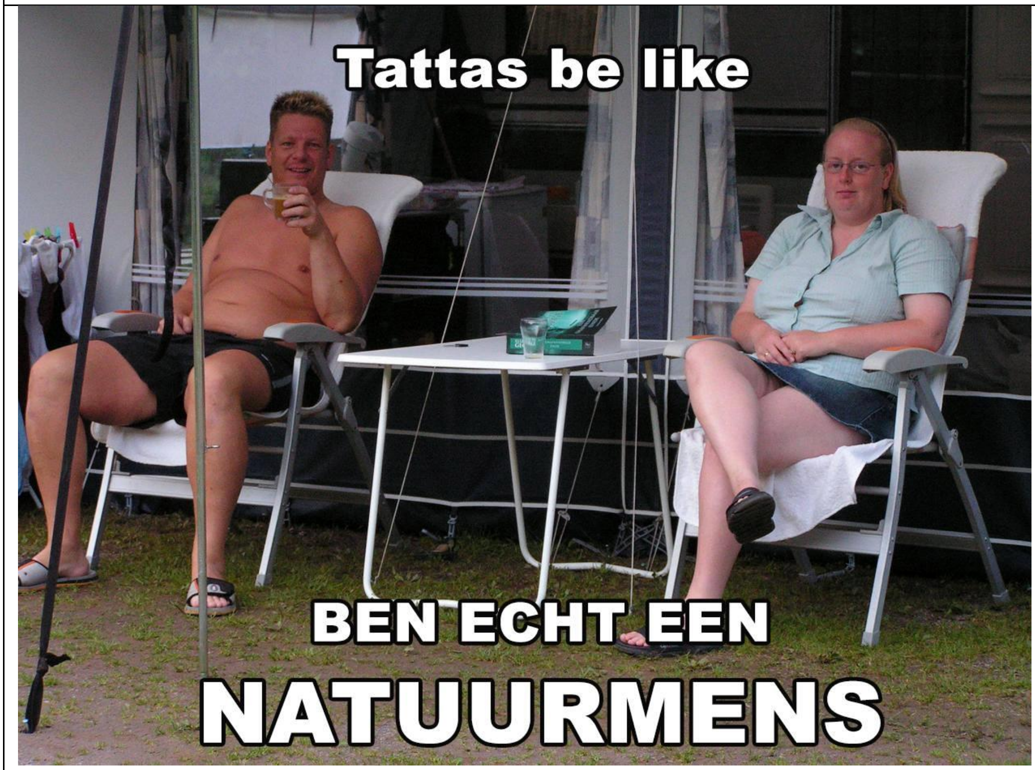

Tatta’s be like: **I am truly a nature person**.

Refers to a very typical Dutch way to go on holiday, namely by going to the same camping every time and setting up your own spot there.

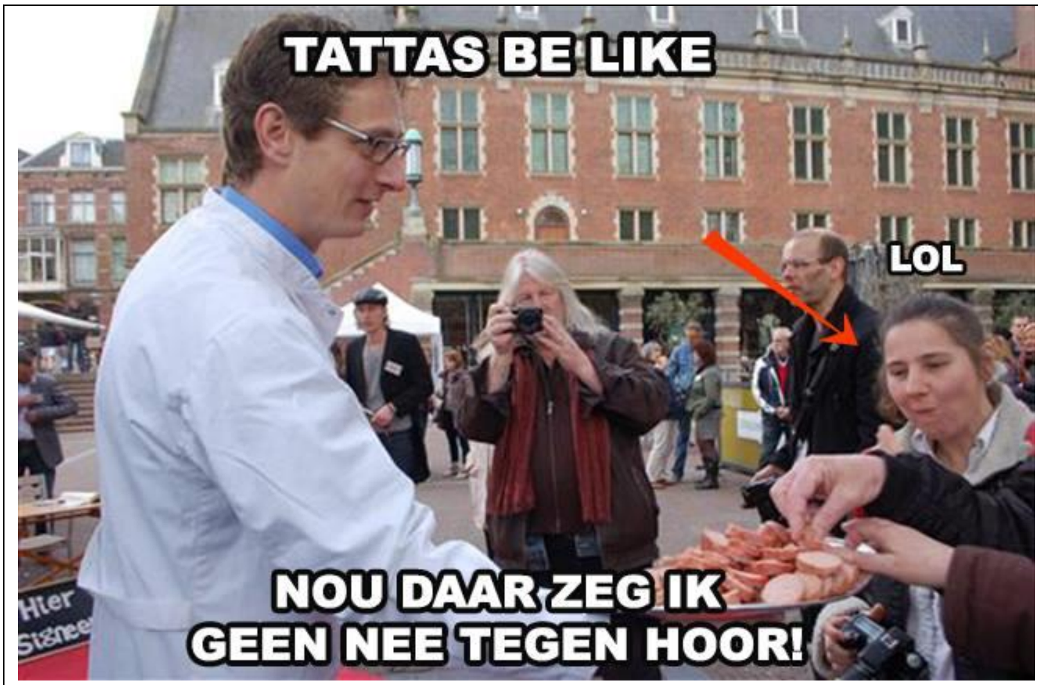

Tatta’s be like: **Well, I won’t say no to that!**

Refers to Dutch people’s love for a traditional snack, liverwurst, especially when it’s being handed out for free.

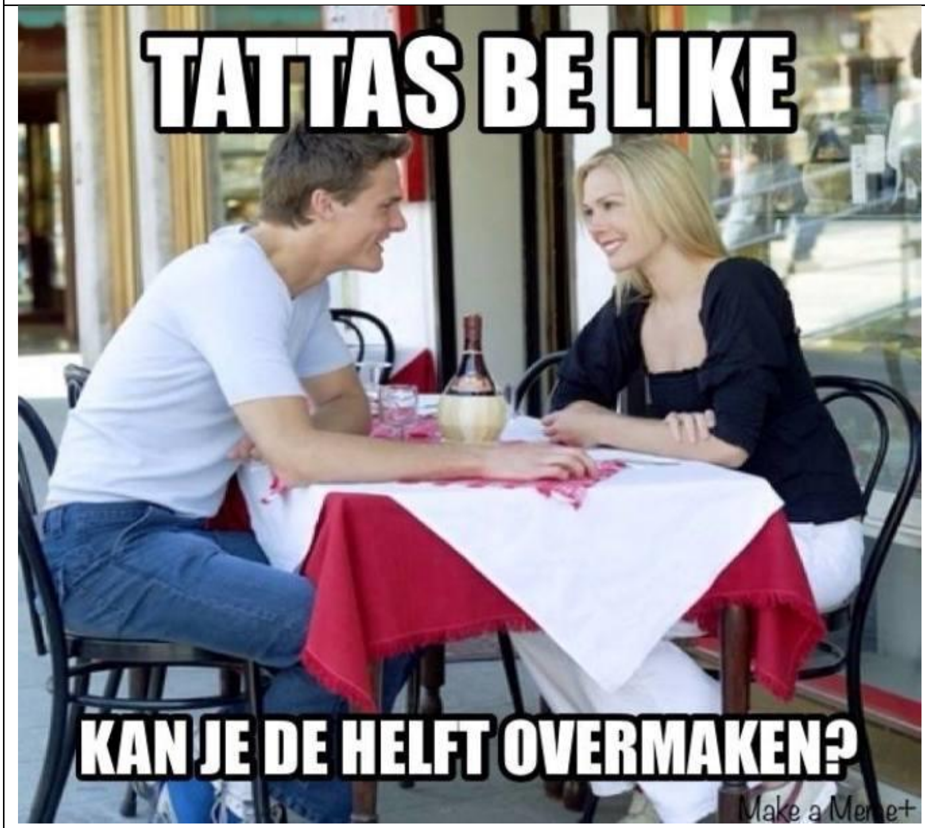

Tatta’s be like: **Could you transfer half (of the bill) to me?**

Refers a Dutch custom to split the bill evenly, even on romantic dates. Also relates to Dutch stinginess.

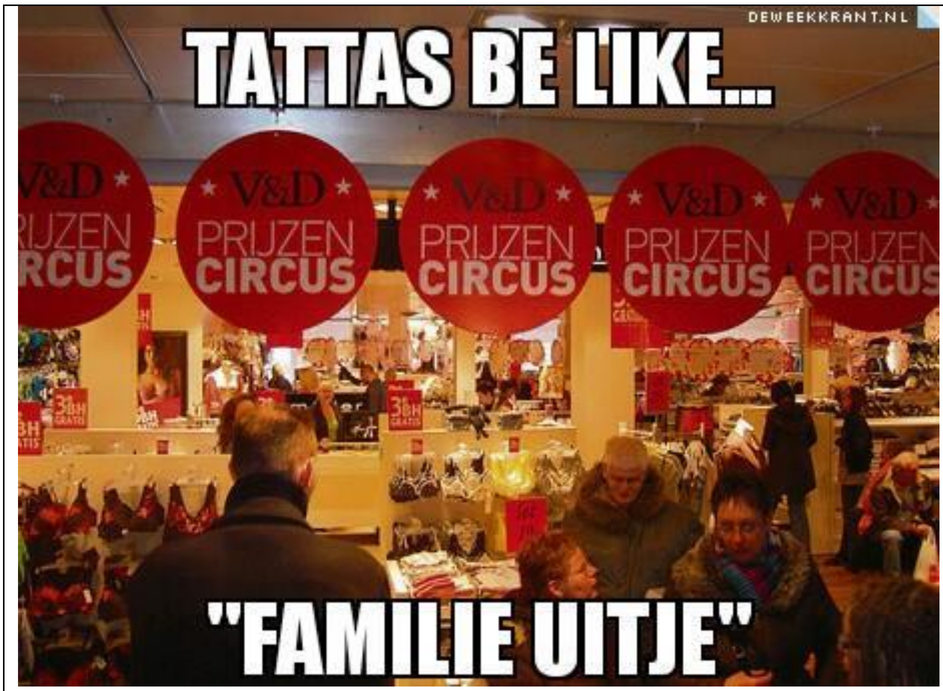

Tatta’s be like: **Family outing**

Refers to a very well-known Dutch department store (V&D) which has a specific sale (Prijzencircus) which attracts a lot of Dutch.

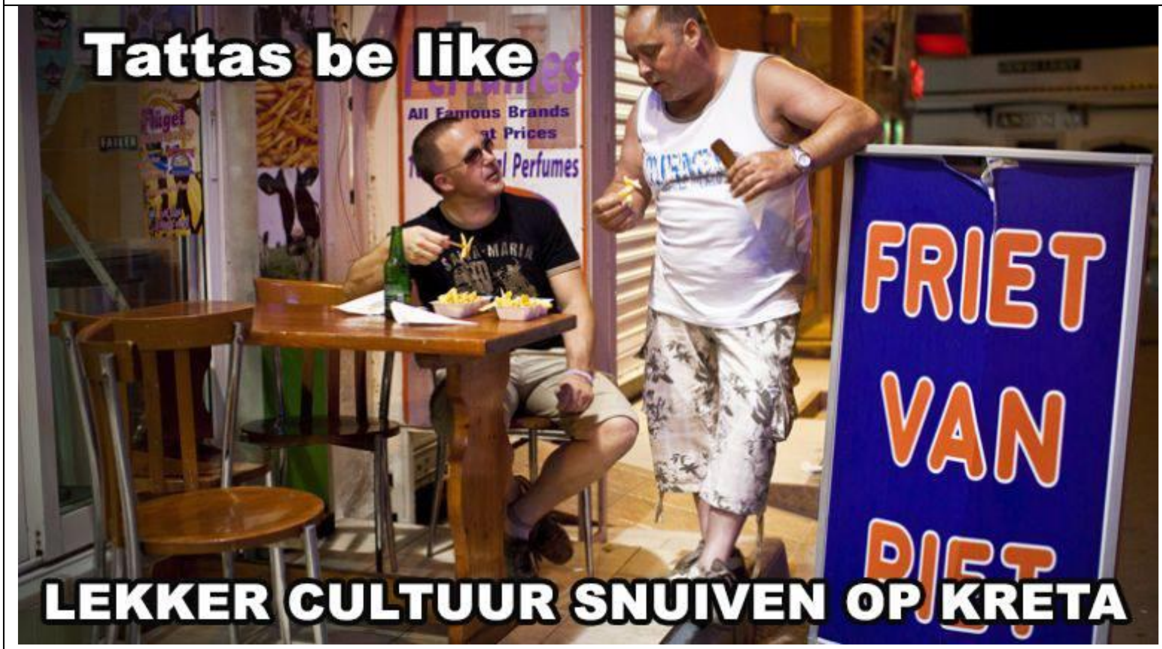

Tatta’s be like: **Having a cultural experience on Crete**

Refers to a popular Dutch tourist destination, Crete, so popular that Dutch people settle there or open a shop for Dutch French fries there.

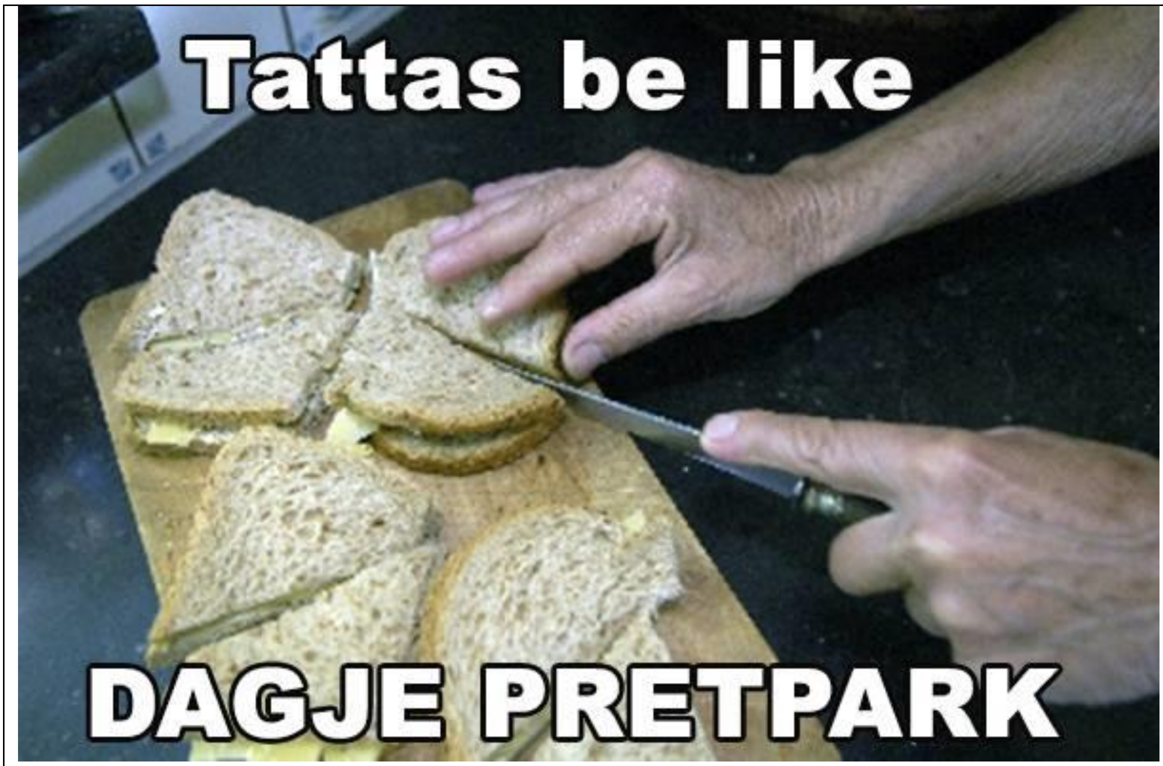

Tatta’s be like: **Outing to the amusement park**

Refers to the Dutch habit of always bringing your own (peanut butter) sandwiches to outings so one doesn’t have to buy food (stinginess).

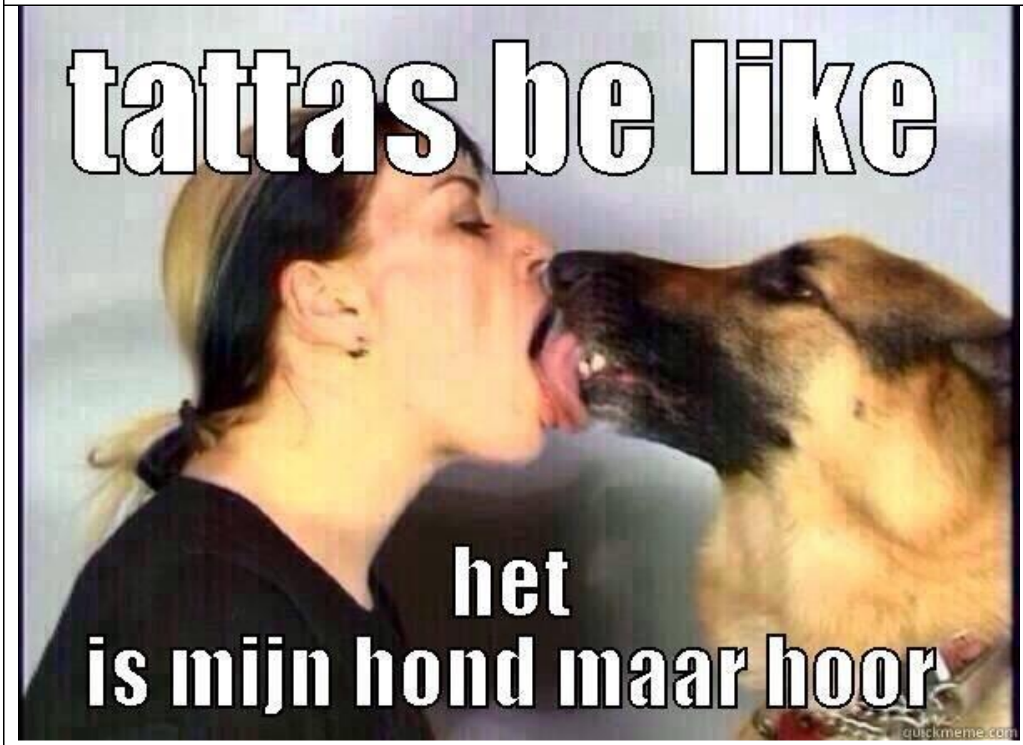

Tatta’s be like: **Oh, it’s just my dog**

Refers to Dutch people doting on their pets, mostly dogs and cats, as if they were their children.

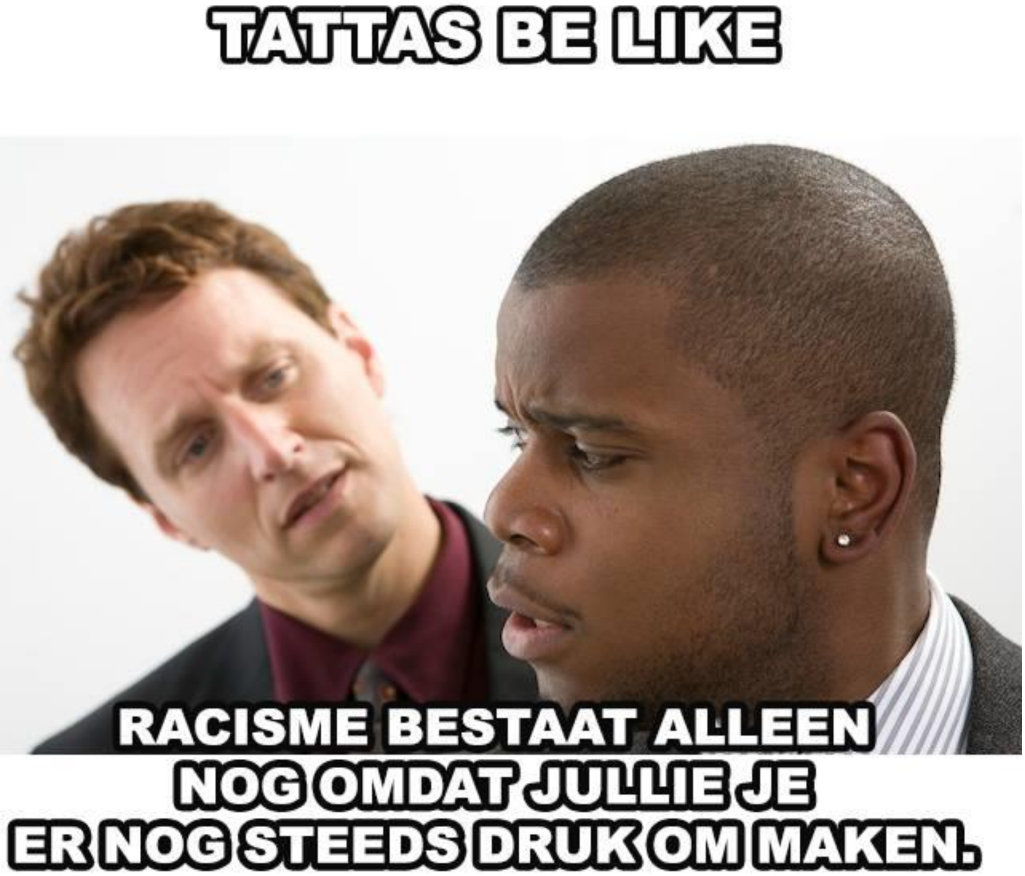
Tatta’s be like: **Racism only exists because you still worry about it**<br>Refers to Dutch people who do not think racism is a big problem
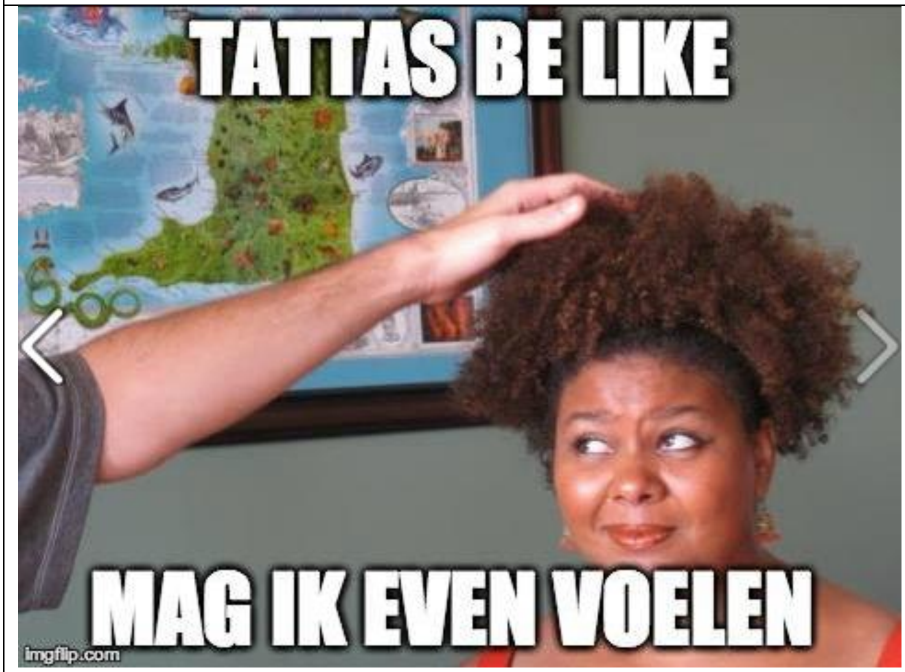
Tatta’s be like: **Can I a feel that for a sec**<br>Refers to Dutch insensitivity to ethnic minorities/other cultures

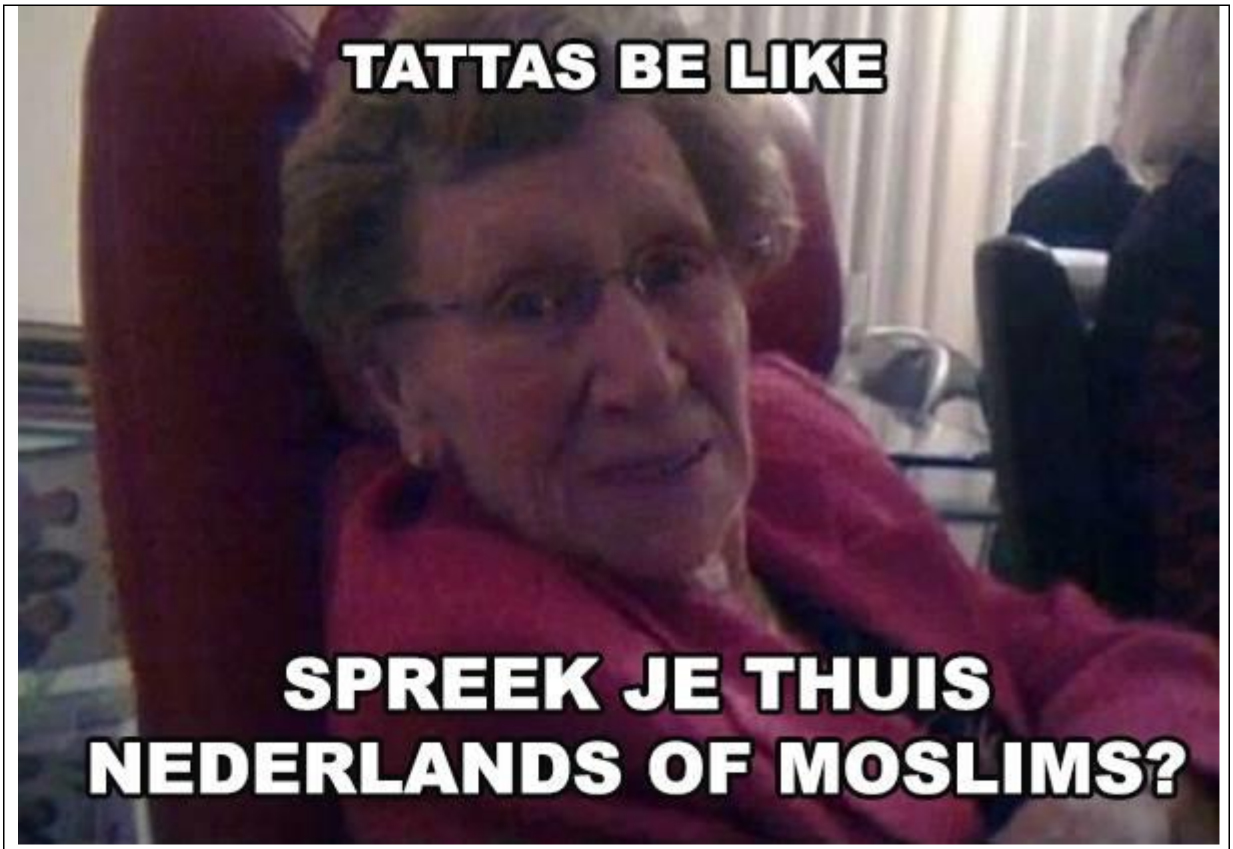

Tatta’s be like: **Do you speak Dutch or Muslim as home?**

Refers to Dutch naivety about ethnic minorities/other cultures

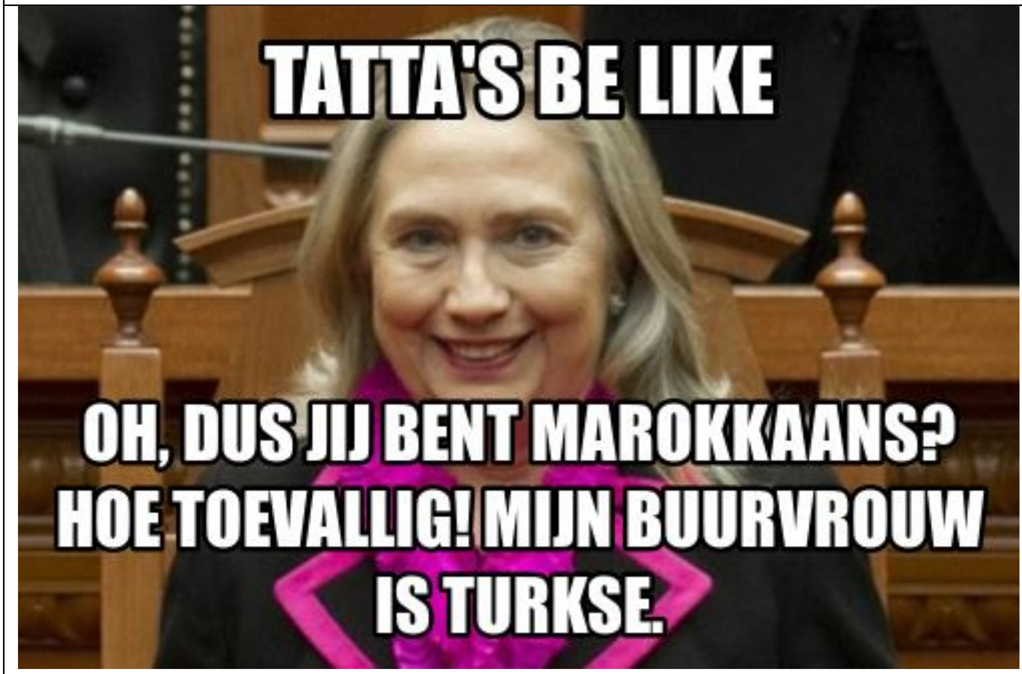

Tatta’s be like: **Oh, you’re Moroccan? What a coincidence, my neighbor is Turkish.**

Refers to Dutch insensitivity/nativity to ethnic minorities/other cultures

Support for Sinterklaas instructions
De laatste paar jaren is er steeds meer ophef geweest over het Sinterklaasfeest in Nederland en met name over de rol van Zwarte Piet. De volgende vragen gaan over de discussie omtrent Sinterklaas en Zwarte Piet. Geef aan of je met de stellingen eens bent.
There has been increasing commotion about the Sinterklaas festivity in the Netherlands, especially about the role of Zwarte Piet. The next questions will be about the discussion surrounding Sinterklaas and Zwarte Piet. Indicate whether you agree with the statements.

Support for Sinterklaas
Geef nu aan in hoeverre je het eens bent met de volgende stellingen op een schaal van: 1 (helemaal wel) tot 9 (helemaal niet)<br><br>- Sinterklaas vieren hoort bij Nederland.<br>- Zwarte Piet is racistisch.<br>- Het uiterlijk van Zwarte Piet moet worden aangepast.<br>- Zwarte Piet kan echt niet meer in deze tijd.<br>- Zwarte Piet moet een onderdeel blijven van het Sinterklaasfeest.<br>- Het Sinterklaasfeest en Zwarte Piet zijn een belangrijk onderdeel van de Nederlandse traditie.<br>- Alleen Nederlanders begrijpen waarom het Sinterklaasfeest belangrijk is.<br>- Sinterklaas is een onschuldig feest voor kinderen.
Indicate whether you agree with the following statements on a scale of: 1 (completely agree) to 9 (completely disagree)<br><br>- Celebrating Sinterklaas is part of the Netherlands<br>- Zwarte Piet (the character) is racist<br>- The appearance of Zwarte Piet should be adjusted<br>- Zwarte Piet (the character) is really not done in today's society<br>- Zwarte Piet should remain a part of the Sinterklaas tradition<br>- Sinterklaas and Zwarte Piet are an important part of Dutch tradition<br>- Only the Dutch understand the value of the Sinterklaas tradition<br>- Sinterklaas is a harmless festivity for children
