# Supplementary material for: Terror Management in a Multicultural Society: Effects of Mortality Salience on Attitudes to Multiculturalism Are Moderated by National Identification and Self-Esteem Among Native Dutch People
Source: Front Psychol. 2018 May 15;9:721. doi: 10.3389/fpsyg.2018.00721 (PMC5962795; doi:10.3389/fpsyg.2018.00721)
Supplement: Supplementary file 2 [file Presentation_2.PDF]

Appendix A: Study 1-2: Exploratory analyses

Appendix B: Study 1: Detailed results per dependent measure

Appendix C: Study 2: Detailed ratings of depictions of Dutch stereotypes

Appendix D: Factor Analyses of the “Tattas” Measure and the Self-stereotyping Measure

Appendix E: Methods, Results and (short) Discussion of an Online Replication Attempt among 203 Students at the Vrije Universiteit Amsterdam

Appendix A

Study 1: Exploratory analyses

Warmth ratings for atheists. Unexpectedly, we also found an interaction between mortality salience, self-esteem, and identification on the warmth ratings for atheists, β = -.19, t(130) = -2.15, p = .033, 95% CI [-8.55, -.36].

To interpret the interaction, we derived predicted means for the eight cells crossing self-esteem (± 1 *SD*) and identification (± 1 *SD*) with mortality salience condition (Aiken & West, 1991). As shown in Figure A1, there were no significant effects among participants with high self-esteem, *ts* < 1.24, *ps* > .218. Among participants with low self-esteem, there was a marginally significant interaction between identification and mortality salience, β = .22, t(130) = 1.66, p = .100, 95% CI [-1.07, 12.15].

Simple-slopes analyses showed that under mortality salience, identification increased feelings of warmth towards atheists among individuals with low self-esteem, but the effect was not significant, β = .32, t(130) = 1.53, p = .129, 95% CI [-2.35, 18.25]. Under dentist salience, individuals with low self-esteem were not influenced by identification, β = -.13, t(130) = -.75, p = .456, 95% CI [-11.43, 5.16].

Figure A1. Feeling thermometer ratings for Atheists as a function of identification and mortality salience. Results are shown separately for participants with (a) low self-esteem and (b) high self-esteem. Low and high levels of identification and self-esteem were coded as 1 *SD* below and above the mean, respectively. Feelings of warmth on the thermometer were rated on a scale from 0, *very cold or negative feelings*, to 100, *very warm and positive feelings.*

a. Low self-esteem

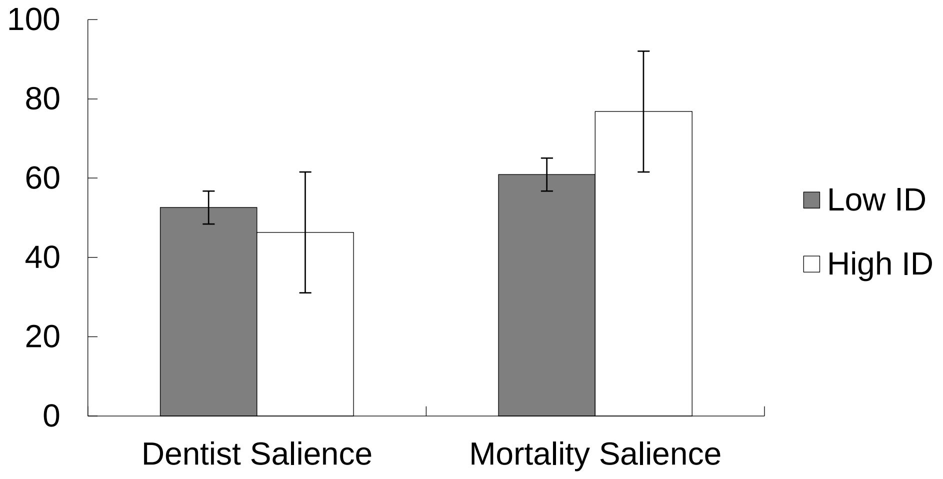

b. High self-esteem

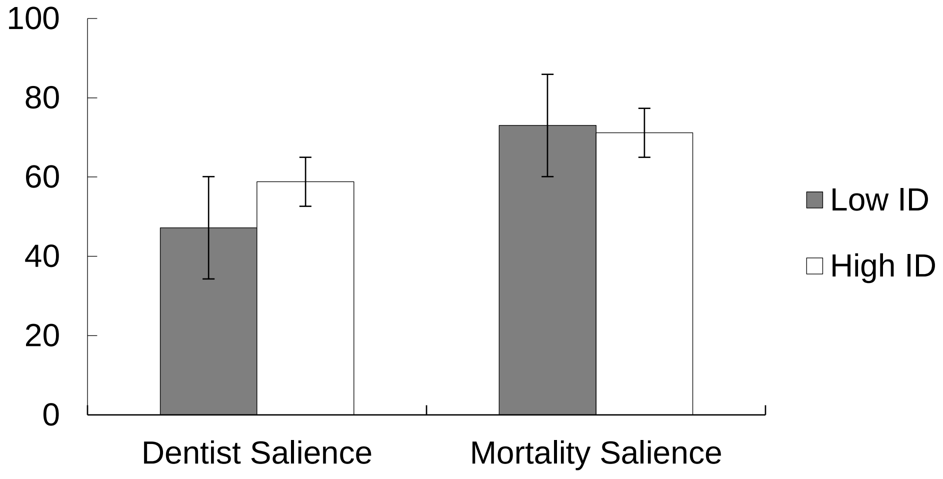

The three-way interaction was not significant for warmth ratings of Jews and Christians, *ts* < .66, *ps* > 509. We did find an interaction between self-esteem and mortality salience on the warmth ratings for Christians, β = .18, *t*(134) = 2.10, *p* = .038, 95% CI [.20, 7.01]. Simple-slopes analyses showed that under mortality salience, low self-esteem individuals reported decreased feelings of warmth towards Christians, β = .30, *t*(130) = 2.58, *p* = .011, 95% CI [1.41, 10.70]. Under dentist salience, self-esteem had no effect, β = -.06, *t*(130) = -.46, *p* = .647, 95% CI [-6.13, 3.82].

Exploratory analyses with the Reading the Mind in the Eyes Task. We also found an interaction between mortality salience, self-esteem, and identification on the Reading the Mind in the Eyes task, β = .29, *t*(130) = 3.40, *p* = .001, 95% CI [.44, 1.68].

To interpret the interaction, we derived predicted means for the eight cells crossing self-esteem (± 1 *SD*) and identification (± 1 *SD*) with mortality salience condition (Aiken & West, 1991). As shown in Figure A2, there was a marginally significant interaction between identification and mortality salience among participants with high self-esteem, β = .21, *t*(130)= 1.97, *p* = .051, 95% CI [-.003, 1.61]. However, subsequent simple-slopes analyses were not significant.

Among participants with low self-esteem, there was a significant interaction between identification and mortality salience, β = -.34, *t*(130) = 2.61, *p* = .010, 95% CI [-2.31, -.32]. Simple-slopes analyses showed that under dentist salience, identification increased emotion recognition among individuals with low self-esteem, β = .43, *t*(130) = 2.64, *p* = .009, 95% CI [.42, 2.91]. Under mortality salience, individuals with low self-esteem were not influenced by identification, β = -.25, *t*(130) = -1.22, *p* = .224, 95% CI [-2.51, .59].

Figure A2. Emotion recognition as a function of identification and mortality salience. Results are shown separately for participants with (a) low self-esteem and (b) high self-esteem. Low and high levels of identification and self-esteem were coded as 1 *SD* below and above the mean, respectively. Emotion recognition scores range from 0 to 36.

a. Low self-esteem

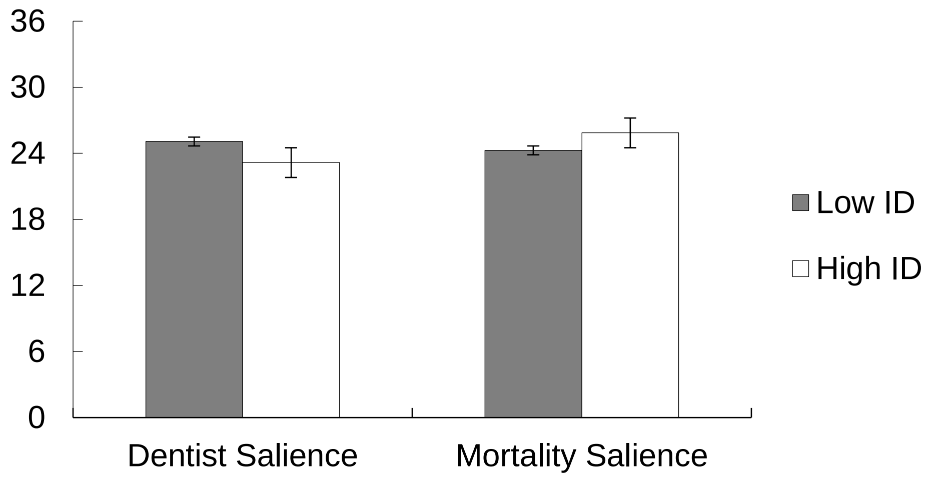

b. High self-esteem

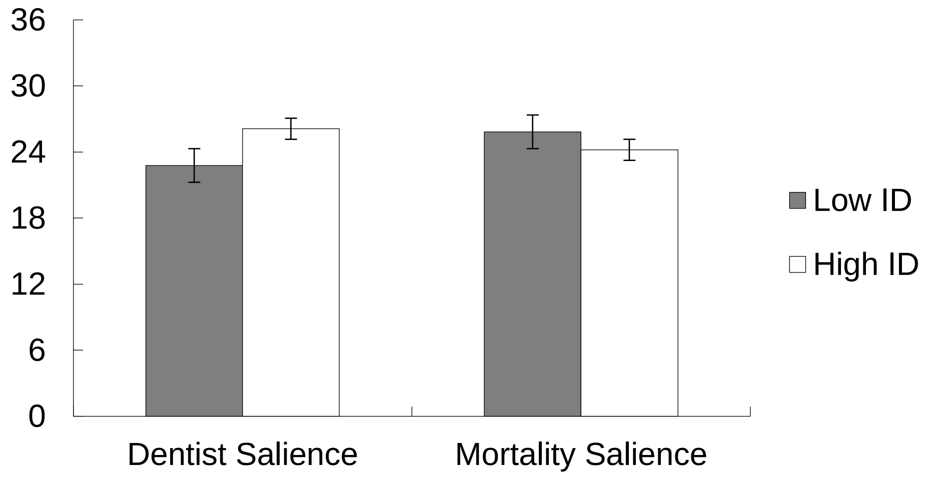

Study 2: Exploratory analyses

Evaluation of Dutch Caricatures. To measure appreciation of caricatures of Dutch people, we selected fifteen images from the Facebook page 'Tattas be like' that mock stereotypical behavior of Dutch people. We asked participants to rate each image on how funny it was on a scale of 1 (*not at all*) to 7 (*very*) (Cronbach's α = .84). We presented the study as being about 'internet humor', and explained that the images were made by ethnic minorities in the Netherlands to make fun of stereotypes and customs of the Dutch people.

Self-stereotyping. We derived eight stereotypes about Dutch people that were depicted as caricatures, namely 'not understanding other cultures', 'loving animals', 'cheap trips', 'going on vacation to places with many Dutch', 'boring birthdays', 'being cheap', 'bad taste in music' and 'hardworking to a fault'. We asked participants to rate per stereotype whether they were characteristic of Dutch people in general (Cronbach's α = .60), and whether they were characteristic of themselves on a scale of 1 (*not at all*) to 7 (*very*) (Cronbach's α = .44).

Results

Appreciation of Dutch caricatures

To test whether people low on national identification with low self-esteem would be more appreciative of the Dutch caricatures under mortality salience, we coded and standardized the independent variables, computed interaction terms and entered the same factors into a multiple regression analysis as in Study 1. The analysis yielded a significant three-way interaction between mortality salience, national identification, and self-esteem β = .17, $t(166) = 2.25$, p = .026, 95% CI [.02, .31]. This interaction is visually displayed in Figure A3.

To interpret the interaction, we derived predicted means for the eight cells crossing self-esteem (± 1 *SD*) and identification (± 1 *SD*) with mortality salience condition (Aiken & West,

1991). There were no effects among participants high on national identification, *t*s < .87, *p*s > .388 (see Figure A3). In contrast, we found a significant interaction between self-esteem and mortality salience among participants low on national identification, β = -.27, *t*(166) = -2.54, *p* = .012, 95% CI [-.46, -.06]. Simple-slopes analysis showed that mortality salience had no effect on those low on national identification with high self-esteem, β = -.24, *t*(166) = -1.55, *p* = .123, 95% CI [-.52, .06]. Among people low on national identification with low self-esteem, mortality salience led to higher perceived funniness of the caricatures, β = .30, *t*(166) = 2.03, *p* = .044, 95% CI [.01, .56]. However, alternative simple slope analyses also found that participants in the dentist salience condition showed an effect of identification, such that participants low on national identification reported a decrease in appreciation of the caricatures in the dentist salience condition, β = .36, *t*(166) = 2.23, *p* = .027, 95% CI [.04, .65]. In the mortality salience condition, identification was not associated with appreciation of the caricatures, β = -.09, *t*(166) = -.16, *p* = .542.

Figure A3 Appreciation of Dutch caricatures as a function of mortality salience and self-esteem among participants a) high versus b) low on national identification.

a. High National Identification

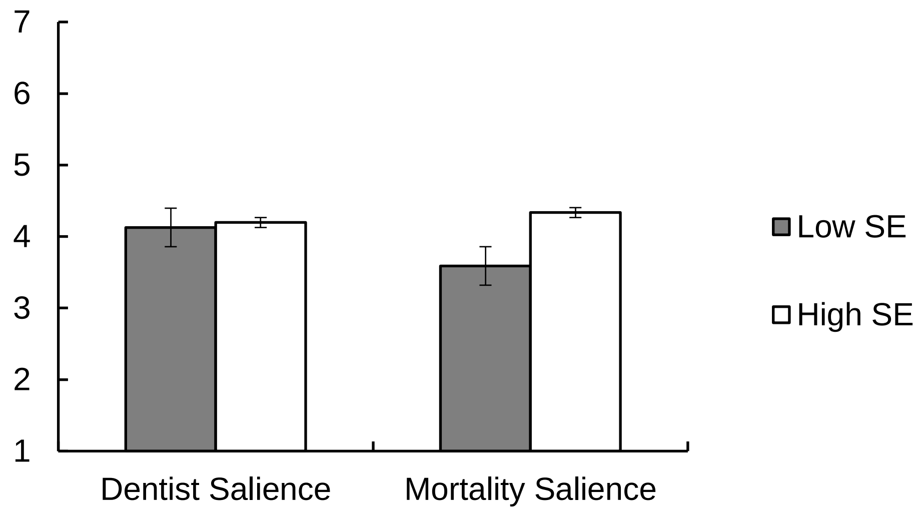

b. Low National Identification

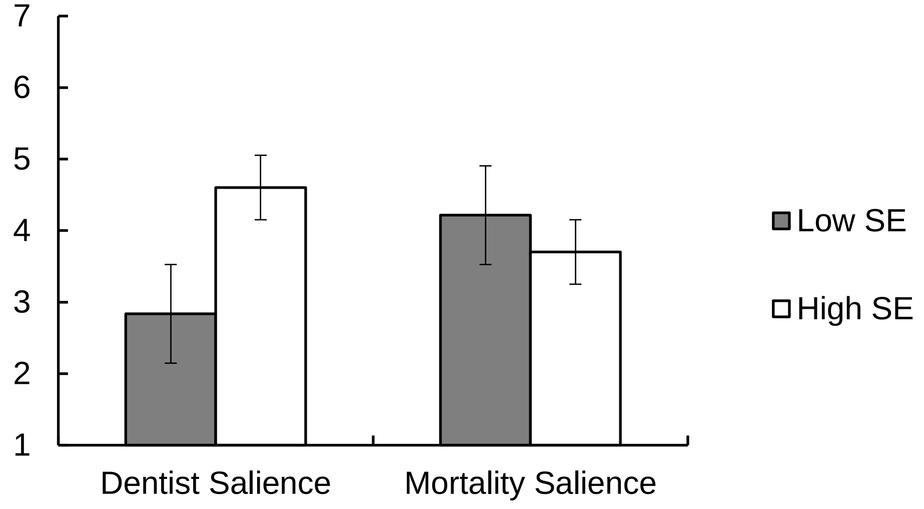

Note: Low and high levels of national identification, mortality salience and self-esteem were coded as 1 *SD* below and above the mean, respectively. The images were rated on a scale from 1, *not at all funny*, to 7, *very funny*. Standard errors are shown as error bars attached to each column.

Self-stereotyping. We then tested whether people with low self-esteem and low national identification would be more likely to self-stereotype under mortality salience. The regression analysis yielded a significant effect of self-esteem, β = -.27, $t(166)$ = -3.72, p < .001, 95% CI [-.30, -.09], national identification, β = .19, $t(166)$ = 2.67, p = .008, 95% CI [.04, .24], an interaction between self-esteem and national identification, β = .16, $t(166)$ = 2.28, p = .024, 95% CI [.02, .23], and a significant three-way interaction between mortality salience, self-esteem and national identification, β = .16, $t(166)$ = 2.30, p = .023, 95% CI [.02, .23]. This interaction effect is visually displayed in Figure A4.

To interpret the interaction, we derived predicted means for the eight cells crossing self-esteem (± 1 *SD*) and identification (± 1 *SD*) with mortality salience condition (Aiken & West,

1991).There were no effects among participants high on national identification, *β* = .16, *t*(166) = 1.52, *p* = .130, 95% CI [-.03, .27]. By contrast, among participants low on national identification, there were was a main effect of self-esteem, *β* = -.43, *t*(166) = -4.34, *p* > .001, 95% CI [-.46, -.17], and a marginal interaction between mortality salience and self-esteem, *β* = -.18, *t*(166) = -2.21, *p* = .080, 95% CI [-.27, .02].

Simple slopes analyses showed that, in the dentist salience condition, self-esteem was non-significantly negatively associated with self-stereotyping among participants low on national identification, *β* = -.26, *t*(166) = -1.79, *p* = .075, 95% CI [-.39, .02]. However, participants low on national identification showed a highly significant negative association between self-esteem and self-stereotyping in the mortality salience condition, such that participants low on national identification with low self-esteem showed more self-stereotyping, *β* = -.61, *t*(166) = -4.39, *p* < .001, 95% CI [-.64, -.24].

This pattern of findings further support that a lowering of the status of Dutch culture allowed participants low on national identification with low self-esteem to view themselves as stereotypically Dutch under mortality salience, while those who identify with Dutch culture for self-enhancement purposes were not affected by mortality saliences. Indeed, if anything, the latter group became somewhat less likely to self-stereotype, although this pattern was not statistically significant.

Figure A4. Self-stereotyping as a function of mortality salience and self-esteem among participants a) high versus b) low on identification.

a. High National Identification

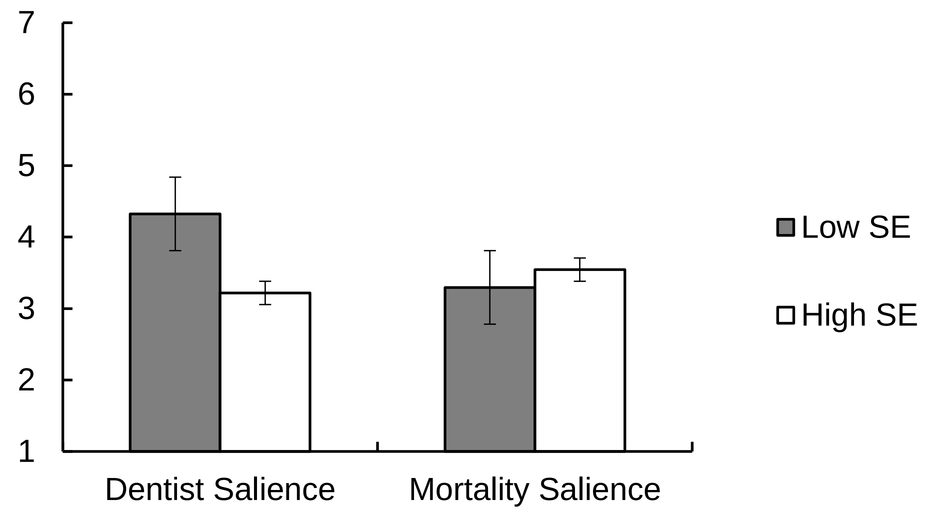

b. Low National Identification

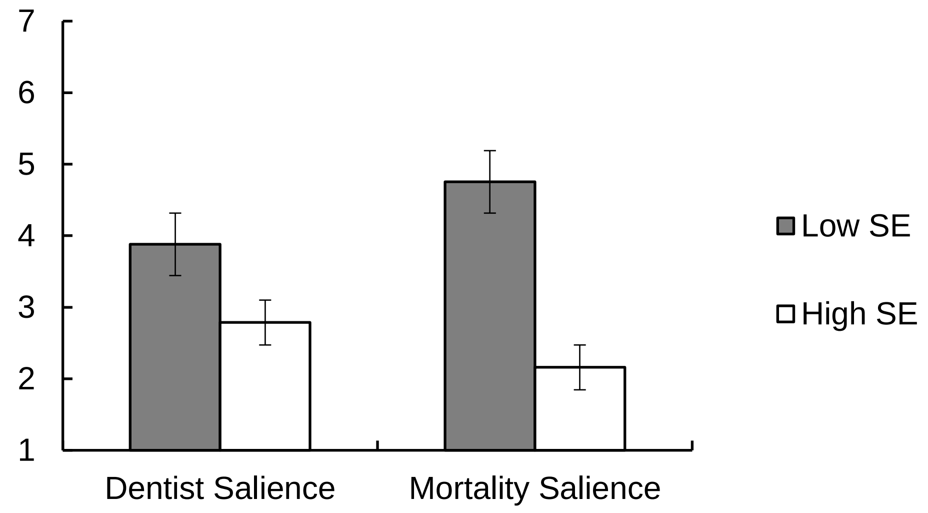

Note: Low and high levels of identification and self-esteem were coded as 1 *SD* below and above the mean, respectively. The Dutch stereotypes were rated on a scale from 1, *not typical of self*, to 7, *very typical of self*. Standard errors are shown as error bars attached to each column.

Appendix B

Study 1: Detailed results per dependent measure

Table B1. Overview of results of the multiple regression and subsequent simple slope analyses per dependent variable

Effect type	Effect	*B*	*T*	*P*	CI	95%
Warmth ratings for Muslims						
Main	MS	-.06	-.70	.485	-4.86	2.32
	**SE**	**.24**	**2.92**	**.004**	**1.72**	**8.94**
	**ID**	**-.23**	**-2.67**	**.009**	**-8.81**	**-1.31**
Interaction	MS x SE	.10	1.22	.224	-1.38	5.84
	MS x ID	-.06	-.71	.480	-5.09	2.41
	SE x ID	.10	1.20	.232	-1.37	5.58
	**MS x SE x ID**	**.18**	**2.06**	**.041**	**.15**	**7.10**
Simple slopes: Low SE	**MS x ID**	**-.22**	**-1.75**	**.082**	**-10.58**	**.64**
Simple slopes: High SE	MS x ID	.10	.99	.323	-2.27	6.85
Simple slopes: Low SE, High MS	**ID**	**-.54**	**-2.75**	**.007**	**-20.88**	**-3.40**
Simple slopes: Low SE, Low MS	ID	-.10	-.62	.537	-9.24	4.84
Ethnocentric emotions						
Main	MS	.03	.39	.700	-.30	.45
	SE	.07	.87	.385	-.21	.54
	**ID**	**-.25**	**-2.95**	**.004**	**-.98**	**-.19**
Interaction	MS x SE	.08	.93	.355	-.20	.56
	**MS x ID**	**.16**	**1.88**	**.062**	**-.02**	**.77**

	SE x ID	.10	1.16	.250	-.15	.58
	MS x SE x ID	.05	.60	.549	-.25	.47
Evaluation of anti-Dutch essay						
Main	MS	.02	.18	.854	-.19	.23
	SE	.02	.27	.788	-.18	.24
	**ID**	**-.20**	**-2.31**	**.023**	**-.48**	**-.04**
Interaction	MS x SE	.05	.62	.534	-.15	.28
	MS x ID	.02	.22	.824	-.20	.25
	SE x ID	-.07	-.84	.404	-.29	.12
	**MS x SE x ID**	**.21**	**2.45**	**.016**	**.05**	**.46**
Simple slopes: Low SE	MS x ID	-.18	-1.37	.173	-.56	.10
Simple slopes: High SE	**MS x ID**	**.22**	**2.05**	**.042**	**.01**	**.55**
Simple slopes: High SE, High MS	ID	-.05	-.38	.707	-.41	.28
Simple slopes: High SE, Low MS	**ID**	**-.49**	**-3.00**	**.003**	**-1.04**	**-.21**
Acceptance of ethnic minorities in Dutch society						
Main	MS	.08	1.03	.304	-.08	.26
	SE	.26	3.49	**.001**	.13	.48
	ID	-.34	-4.52	**<.001**	-.59	-.23
Interaction	MS x SE	.17	2.35	**.020**	.03	.38
	MS x ID	-.09	-1.17	.243	-.29	.07
	SE x ID	-.11	-1.41	.160	-.28	.05
	MS x SE x ID	.27	3.59	**<.001**	.14	.47
**Simple slopes: Low SE**	**MS x ID**	**-.34**	**-3.01**	**.003**	**-.68**	**-.14**

**Simple slopes: High SE**	**MS x ID**	**.16**	**1.77**	**.079**	**-.02**	**.41**
**Simple slopes: Low SE, High MS**	**ID**	**-.60**	**-3.31**	**.001**	**-1.11**	**-.28**
Simple slopes: Low SE, Low MS	ID	.10	.69	.493	-.22	.45
Acceptance of Muslims in Dutch society						
Main	MS	.03	.33	.743	-.22	.31
	**SE**	**.16**	**2.05**	**.042**	**.01**	**.54**
	**ID**	**-.31**	**-3.76**	**.000**	**-.80**	**-.25**
Interaction	MS x SE	.10	1.29	.200	-.09	.44
	MS x ID	-.09	-1.07	.287	-.43	.13
	SE x ID	-.08	-1.00	.319	-.39	.13
	**MS x SE x ID**	**.24**	**2.97**	**.004**	**.13**	**.64**
**Simple slopes: Low SE**	**MS x ID**	**-.31**	**-2.56**	**.012**	**-.95**	**-.12**
Simple slopes: High SE	MS x ID	.14	1.39	.168	-.10	.57
**Simple slopes: Low SE, High MS**	**ID**	**-.55**	**-2.85**	**.005**	**-1.57**	**-.29**
Simple slopes: Low SE, Low MS	ID	.08	.53	.597	-.38	.66

Warmth ratings for Muslims. The analysis yielded an effect of self-esteem, β = .24, t(130) = 2.92, p = .004, 95% CI [1.72, 8.94], an effect of identification, β = -.23, t(130) = -2.67, p = .009, 95% CI [-8.81, -1.31], and the predicted interaction between mortality salience, self-esteem, and identification, β = .18, t(130) = 2.06, p = .041, 95% CI [.15, 7.10].

To interpret the interaction, we derived predicted means for the eight cells crossing self-esteem (± 1 *SD*) and identification (± 1 *SD*) with mortality salience condition (Aiken & West, 1991). As shown in Figure B1, there were no significant effects among participants with high self-esteem, *ts* < 1.28, *ps* > .202. Among participants with low self-esteem, there was a significant main effect of identification, β = -.32, t(130) = -2.53, p = .013, 95% CI [-12.78, -1.56], qualified by a marginally significant interaction between identification and mortality salience, β = -.22, t(130) = -1.75, p = .082, 95% CI [-12.78, -1.56].

Simple-slopes analyses showed that under mortality salience, identification decreased feelings of warmth towards Muslims among individuals with low self-esteem, β = -.54, t(130) = -2.75, p = .007, 95% CI [-20.88, -3.40]. Under dentist salience, individuals with low self-esteem were not influenced by identification, β = -.10, t(130) = -.62, p = .537, 95% CI [-9.2, 4.84].

Figure B1. Feeling thermometer ratings for Muslims as a function of identification and mortality salience. Results are shown separately for participants with (a) low self-esteem and (b) high self-esteem. Low and high levels of identification and self-esteem were coded as 1 *SD* below and above the mean, respectively. Feelings of warmth on the thermometer were rated on a scale from 0, *very cold or negative feelings*, to 100, *very warm and positive feelings.*

a. Low self-esteem

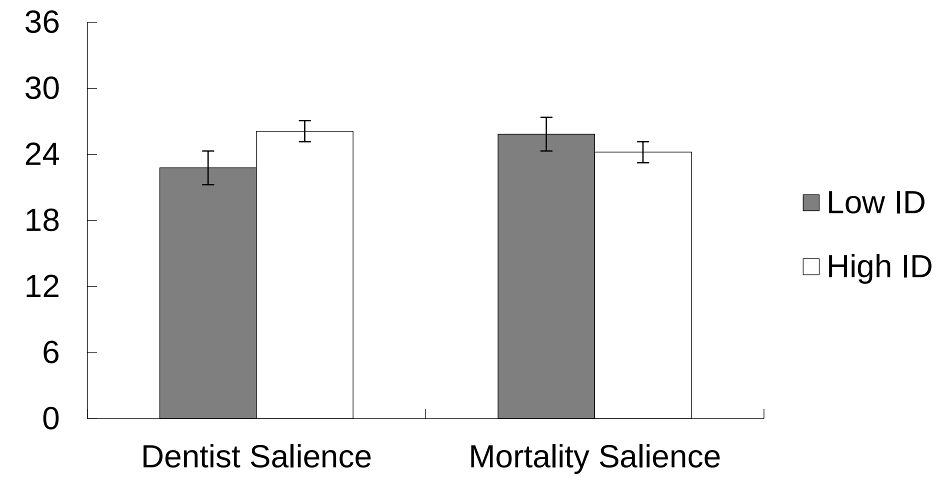

b. High self-esteem

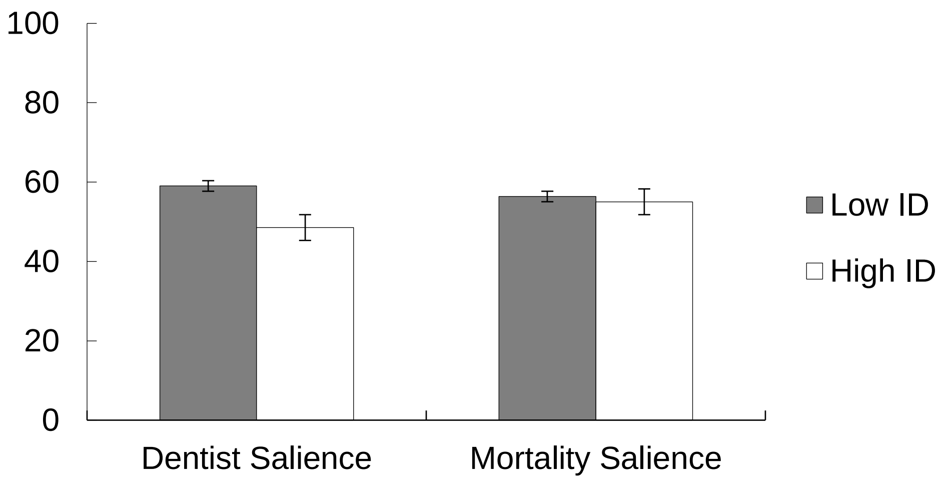

Ethnocentric emotions. The multiple regression analysis yielded an effect of identification, β = -.25, t(130) = -2.95, p = .004, 95% CI [-.98, -.19], and a marginally significant interaction between mortality salience and identification, β = .16, t(130) = 1.88, p = .062, 95% CI [-.02, .77]. The predicted interaction between mortality salience, self-esteem, and identification was not significant, β = .05, t(130) = .60, p = .549, 95% CI [-.53, .47].

We entered mortality salience and identification into a multiple regression analysis predicting ethnocentrism. As shown in Figure B2, the analysis yielded an interaction effect between mortality salience and identification, β = .19, t(134) = 2.27, p = .025, 95% CI [.06, .81]. We derived predicted means for the four cells crossing identification (± 1 *SD*) with mortality salience condition (Aiken & West, 1991). Simple-slopes analyses showed that under dentist salience, identification increased ethnocentrism, β = -.42, t(134) = -3.52, p = .001, 95% CI [-1.49, .-42]. Under mortality salience, there was no effect of identification such that high and low identifiers were equally ethnocentric, β = -.04, t(134) = -.37, p = .716, 95% CI [-.62, .43].

Figure B2. Ethnocentric emotions as a function of identification and mortality salience. Low and high levels of identification and mortality salience were coded as 1 *SD* below and above the mean, respectively. The ethnocentric emotions index could theoretically range from -16 (maximum pro-Dutch/anti-Muslim bias) to +16 (maximum anti-Dutch/pro-Muslim bias).

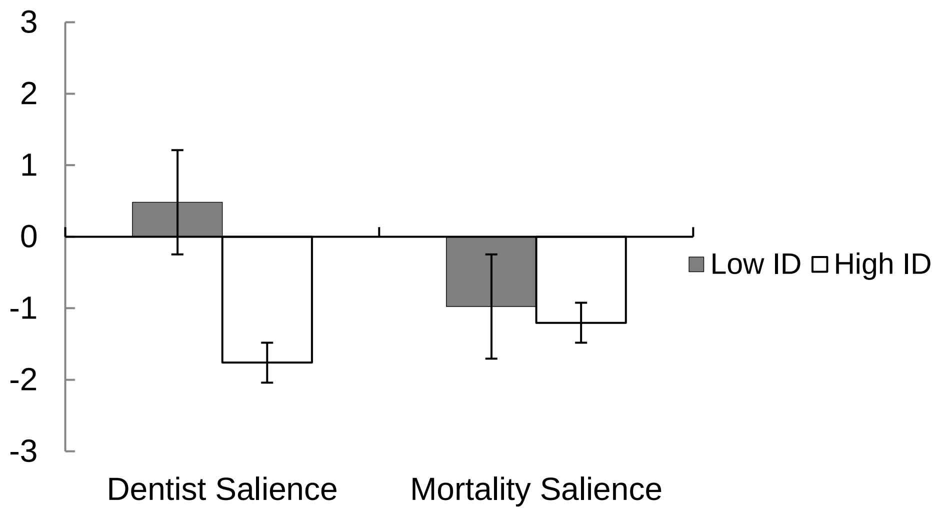

Evaluation of the anti-Dutch essay. We ran the same analysis for the evaluations of the pro-Dutch and anti-Dutch essay that were ostensibly written by international students from Morocco. There were no interaction effects for the pro-Dutch essay, $ts < 1.30$, $ps < .197$. For the anti-Dutch essay, the regression analysis yielded an effect of identification, $\beta = -.20$, $t(130) = -2.31$, $p = .023$, 95% CI [-.48, -.04], and the predicted interaction between existential threat, self-esteem, and identification, $\beta = .21$, $t(130) = 2.45$, $p = .016$, 95% CI [.05, .46].

To interpret the interaction, we derived predicted means for the eight cells crossing self-esteem (± 1 *SD*) and identification (± 1 *SD*) with existential threat condition (Aiken & West, 1991). As shown in Figure B3 there were no significant effects among participants with low self-esteem, $ts < 1.37$, $ps > .173$. Among participants with high self-esteem, there was a significant interaction between identification and existential threat, $\beta = .26$, $t(130) = 2.05$, $p = .042$, 95% CI [.01, .55].

Simple-slopes analyses showed that among participants with high self-esteem, in the low mortality salience condition, identification was associated with more negative evaluations of the anti-Dutch essay, $\beta = -.488$, $t(130) = -3.00$, $p = .003$, 95% CI [-1.04, -.21]. In the mortality salience condition, identification had no effect, $\beta = -.05$, $t(130) = -.38$, $p = .707$, 95% CI [-.41, .28].

We also reran these analyses separately for item type (items about essay writer versus items about the essay itself). We found a marginal three-way interaction for the essay items, $\beta = .239$, $t(130) = 1.750$, $p = .082$, while the three-way for items targeting the writer was highly significant, $\beta = .26$, $t(130) = 3.03$, $p = .003$, 95% CI [.09, .45]. These exploratory analyses suggest that participants with high self-esteem were more negative specifically in their evaluation of the (ostensibly Morrocan) essay writer in the control condition.

Figure B3. Evaluation of the anti-Dutch essay as a function of identification and mortality salience. Results are shown separately for participants who scored (a) low and (b) high on self-esteem. Low and high levels of identification and self-esteem were coded as 1 *SD* below and above the mean, respectively. The evaluation items were rated on a scale from 1 to 9.

a. Low self-esteem

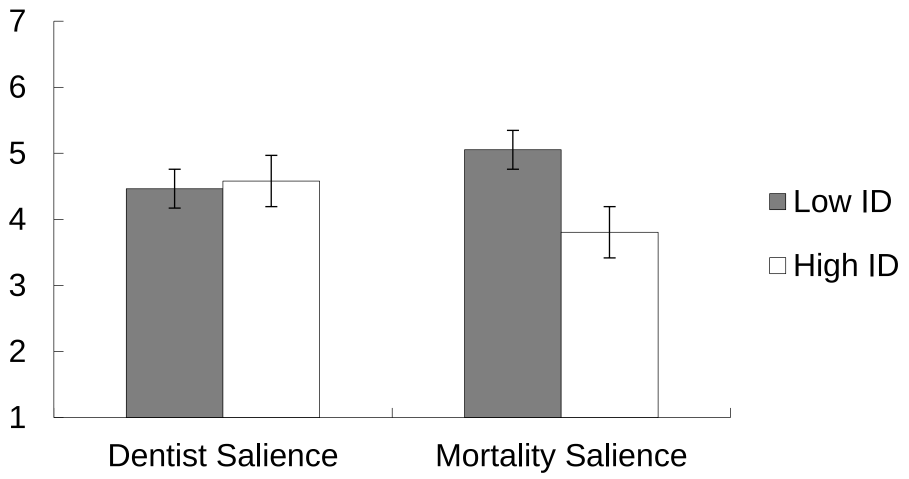

b. High self-esteem

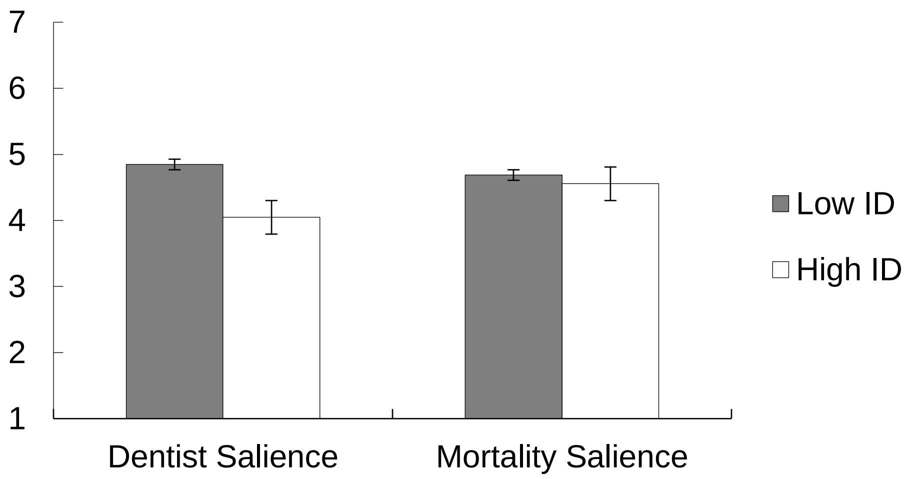

Acceptance of ethnic minorities in Dutch society. The analysis yielded an effect of self-esteem, $\beta = .26$, $t(130) = 3.49$, $p = .001$, 95% CI [.13, .48], an effect of identification, $\beta = -.34$,

t(130) = -4.52, *p* < .001, 95% CI [-.59, -.23], an interaction between self-esteem and mortality salience, *β* = .17, *t*(130) = 2.35, *p* = .020, 95% CI [.03, .38], and the predicted interaction between mortality salience, self-esteem, and identification, *β* = .273, *t*(130) = 3.590, *p* < .001, 95% CI [.14, .47].

To interpret the interaction, we derived predicted means for the eight cells crossing self-esteem (± 1 *SD*) and identification (± 1 *SD*) with mortality salience condition (Aiken & West, 1991). As shown in Figure B4, among participants with high self-esteem, there was an effect of mortality salience, *β* = .25, *t*(130) = 2.39, *p* = .018, 95% CI [.05, .54], an effect of identification, *β* = -.44, *t*(130) = -4.80, *p* < .001, 95% CI [-.75, -.31], and a marginally significant interaction between mortality salience and identification, *β* = .16, *t*(130) = 1.77, *p* = .079, 95% CI [-.02, .41]. Among participants with low self-esteem, there was a main effect of identification, *β* = -.25, *t*(130) = -2,15, *p* = .034, 95% CI [-.56, -.02], qualified by a significant mortality salience by identification interaction, *β* = -.34, *t*(130) = -3.01, *p* = .003, 95% CI [-.68, -.14]. Simple-slopes analyses showed that under mortality salience, identification decreased acceptance of ethnic minorities among individuals with low self-esteem, *β* = -.59, *t*(130) = -3.31, *p* = .001, 95% CI [-1.11, -.28]. Under dentist salience, individuals with low self-esteem were not influenced by identification, *β* = .10, *t*(130) = .69, *p* = .493, 95% CI [-.22, .45].

Figure B4. Acceptance of ethnic minorities in Dutch society as a function of identification and mortality salience. Results are shown separately for participants with (a) low self-esteem and (b) high self-esteem. Low and high levels of identification and self-esteem were coded as 1 *SD* below and above the mean, respectively. The evaluation items were rated on a scale from 1, *not at all,* to 9, *very much.*

a. Low self-esteem

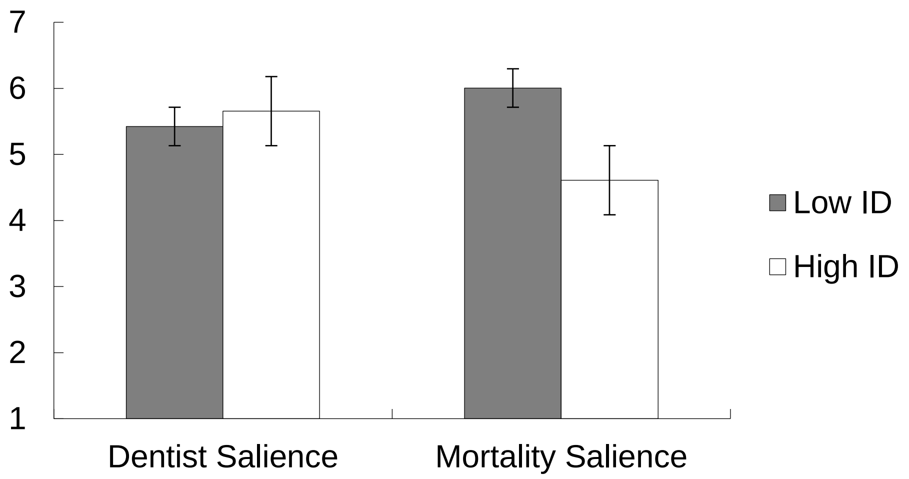

b. High self-esteem

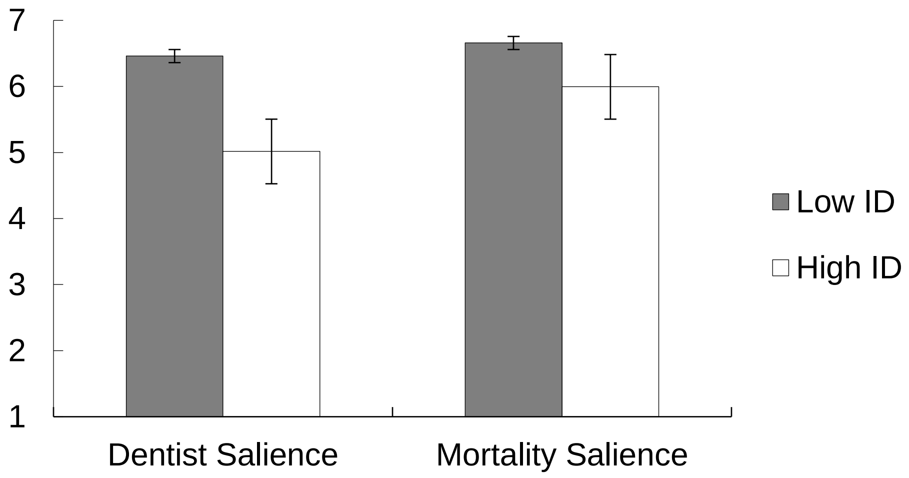

Acceptance of Muslims in Dutch society. The analysis yielded an effect of self-esteem, β = .16, $t(130) = 2.05$, $p = .042$, 95% CI [.01, .54] an effect of identification, β = -.31, $t(130) = -3.76$, $p < .001$, 95% CI [-.80, -.25], and the predicted interaction between mortality salience, self-esteem, and identification, β = .24, $t(130) = 2.97$, $p = .004$, 95% CI [.13, .64].

To interpret the interaction, we derived predicted means for the eight cells crossing self-esteem (± 1 *SD*) and identification (± 1 *SD*) with mortality salience condition (Aiken & West, 1991). As shown in Figure B5, there was a main effect of identification among participants with high self-esteem, β = -.39, $t(130)$ = -3,85, $p < .001$, 95% CI [-.99, -.32], but the interaction between mortality salience and identification was not significant, β = .14, $t(130) = 1.39$, $p = .168$, 95% CI [-.10, .57].

Among participants with low self-esteem, there was a marginally significant main effect of identification, β = -.23, $t(130) = -1.89$, $p = .061$, 95% CI [-.81, .02], qualified by a significant mortality salience by identification interaction, β = -.31, $t(130) = -2.56$, $p = .012$, 95% CI [-.95, -.12]. Simple-slopes analyses showed that under mortality salience, identification decreased acceptance of Muslims among individuals with low self-esteem, β = -.55, $t(130) = -2.85$, $p = .005$, 95% CI [-1.57, -.29]. Under dentist salience, individuals with low self-esteem were not influenced by identification, β = .08, $t(130) = .53$, $p = .597$, 95% CI [-.38, .66].

Figure B5. Acceptance of Muslims as a function of identification and mortality salience.

Acceptance of Muslims as a function of identification and mortality salience. Results are shown separately for participants with (a) low self-esteem and (b) high self-esteem. Low and high levels of identification and self-esteem were coded as 1 *SD* below and above the mean, respectively. Acceptance of ethnic minorities was rated on a scale from 1, *not at all*, to 9, *very much*.

a. Low self-esteem

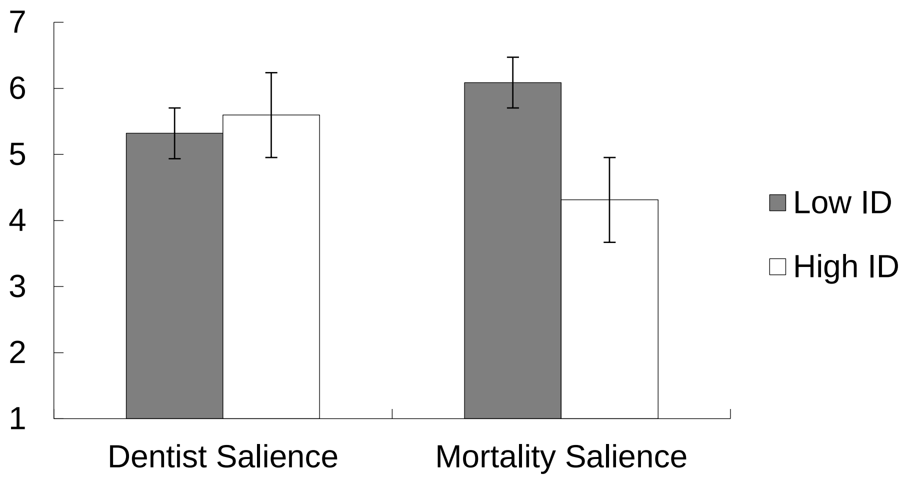

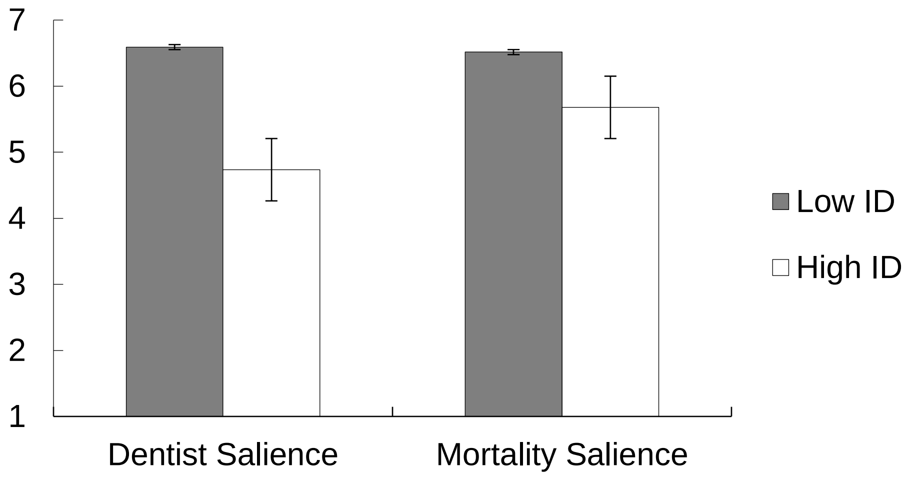

b. High self-esteem

Appendix C

Study 2: Detailed ratings of depictions of Dutch stereotypes

Table C1. Means and standard deviations of the ratings of the stereotypically Dutch images.

	Description of stereotypical depiction and accompanying caption	*M*	*SD*
1	Man and woman dancing at a club with the caption ‘Hardcore is the soundtrack of our lives’	3.79	1.82
2	Person lying in a hospital bed completely covered in bandages with the caption ‘You are still coming to work, right?’	4.24	1.59
3	Birthday celebration where all the guests sit in the living room with the caption ‘Happy birthday with Els’ repeated many times	4.80	1.79
4	Man and woman sitting at a camping site with their trailer with the caption ‘We are truly nature people’	4.41	1.76
5	Woman being overly excited about free ‘liverwurst’ a popular type of sausage with the caption ‘Well, I won’t say no to that!’	4.68	1.70
6	Man and woman on a date with the caption ‘Can you transfer half of the check back to my bank account?’	4.55	1.75
7	A big discount sale of a popular Dutch chain store with the caption ‘Family daytrip’	3.70	1.76
8	Dutch men at a Dutch fast food place in Crete (a popular Dutch travel destination) with the caption ‘Having a cultural experience on Crete’	4.89	1.43
9	Sandwiches with cheese with the caption ‘Day trip to the amusement park'	4.49	1.64
10	Dog licking a woman on the mouth with the caption ‘Oh it’s just my dog!’	3.09	1.99
11	A Dutch man talking to an African-American man with the caption ‘Racism only exists because you are still fussing about it’	2.67	1.64
12	Dutch person touching an African-American woman’s hair with the caption ‘May I touch it’	4.07	1.75
13	An elderly Dutch lady with the caption ‘Do you speak Dutch or ‘Muslim’ at home?’	3.79	1.96

14	An elderly Dutch lady saying with the caption ‘Oh, you’re Moroccan. What a coincidence! My neighbor is Turkish.’	3.40	1.84
15	A Dutch man talking to an African-American woman with the caption ‘I face racism all the time because of my French last name’	3.16	1.75

Table C2. Means and standard deviations of the ratings of how typical these items are of Dutch people and of themselves.

	Typical of Dutch		Typical of self	
Item	*M*	*SD*	*M*	*SD*
Not understanding other cultures	3.82	1.71	2.10	1.20
Loving animals	3.80	1.57	4.45	1.93
Cheap trips	4.86	1.62	3.94	1.79
Going on vacation to places with many Dutch	5.67	1.21	2.66	1.66
Boring birthdays	4.28	1.70	3.24	1.71
Being cheap/stingy	5.28	1.36	3.54	1.69
Bad taste in music	3.37	1.71	2.36	1.43
Hardworking to a fault	4.95	1.29	5.16	1.42

Appendix D: Factor Analyses of the “Tattas” Measure and the Self-stereotyping Measure

To better understand our “Tattas” measure we conducted a principal components factor analysis of the 15 caricatures using varimax (orthogonal) rotations, and looked at whether the images clustered together in an interpretable fashion. Bas sed on initial Eigen values larger than 1.0, four factors were identified, explaining 55.53% of the variance. The factor loading matrix is presented in Table 1. The first and largest component seems to include more general Dutch caricatures, while the rest (11 to 15) loaded high on more than one component, as indicated by the cross-loading above .4. These caricatures had in common that they concerned somewhat controversial jokes about Dutch people making culturally insensitive comments to people with an ethnic background. Internal consistency for the more general Dutch caricatures component using Cronbach’s alpha, was .80, and was high for the culturally insensitive caricatures as well: .71. They were highly correlated, $r = .55$, $p > .001$.

When we repeat the three-way analysis for both components separately, the Dutch caricatures yielded a marginal three-way interaction, $p = .059$, 95% CI [-.01, .31], and the culturally insensitive caricatures yielded a significant three-way interaction between mortality salience, national identification, and self-esteem, $p = .035$, 95% CI [.02, .39]. Thus, results for both factors were similar

Table 1. Factor analysis matrix for “Tattas” measure

		1	2	3	4
1	Man and woman dancing at a club with the caption ‘Hardcore is the soundtrack of our lives’	.52	.05	.13	-.37

2	Person lying in a hospital bed completely covered in bandages with the caption ‘You are still coming to work, right?’	.39	.17	.66	.05
3	Birthday celebration where all the guests sit in the living room with the caption ‘Happy birthday with Els’ repeated many times	.55	-.03	-.30	.30
4	Man and woman sitting at a camping site with their trailer with the caption ‘We are truly nature people’	.66	-.36	-.25	-.17
5	Woman being overly excited about free ‘liverwurst’ a popular type of sausage with the caption ‘Well, I won’t say no to that!’	.60	-.34	-.06	.19
6	Man and woman on a date with the caption ‘Can you transfer half of the check back to my bank account?’	.57	-.28	-.19	.42
7	A big discount sale of a popular Dutch chain store with the caption ‘Family daytrip’	.56	-.30	.28	-.24
8	Dutch men at a Dutch fast food place in Crete (a popular Dutch travel destination) with the caption ‘Having a cultural experience on Crete’	.62	-.26	-.01	.02
9	Sandwiches with cheese with the caption ‘Day trip to the amusement park'	.60	-.34	-.05	-.27

10	Dog licking a woman on the mouth with the caption ‘Oh it’s just my dog!’	.55	.02	.12	-.30
11	A Dutch man talking to an African-American man with the caption ‘Racism only exists because you are still fussing about it’	.46	.42	.09	.25
12	Dutch person touching an African-American woman’s hair with the caption ‘May I touch it’	.43	-.04	.50	.45
13	An elderly Dutch lady with the caption ‘Do you speak Dutch or ‘Muslim’ at home?’	.60	.47	-.12	-.16
14	An elderly Dutch lady saying with the caption ‘Oh, you’re Moroccan. What a coincidence! My neighbor is Turkish.’	.63	.47	-.26	.16
15	A Dutch man talking to an African-American woman with the caption ‘I face racism all the time because of my French last name’	.56	.54	-.11	-.18

We also ran a factor analysis using principal components and varimax (orthogonal) rotations on the 8 descriptors we used for the group-stereotyping and self-stereotyping measures. Group-stereotyping ratings showed a three component solution based on initial Eigen values larger than 1.0, explaining 56.59% of the variance. The factor loadings are presented in Table 2. The first and largest component seems to capture most of the descriptors we used, with the

exception of “Loving animals”, and “Hardworking to a fault”, for which there was presumably less consensus. This component had a Cronbach’s alpha of .67.

Table 2. Factor analysis matrix for group-stereotyping measure

		1	2	3
1	Not understanding other cultures	.57	-.04	.47
2	Loving Animals	.30	.62	.56
3	Cheap trips	.71	.27	-.25
4	Going on vacation to places with many Dutch	.49	.18	-.51
5	Boring birthdays	.56	-.24	.14
6	Being cheap/stingy	.66	-.11	-.33
7	Bad taste in music	.65	-.21	.18
8	Hardworking to a fault	-.13	.73	-.19

For the self-stereotyping measure, we also found a three-component solution based on initial Eigen values larger than 1.0, explaining 54.35% of the variance. The factor loadings are presented in Table 3. The first component seems to refer to descriptors that describe Dutch people as “cheap simpletons”, e.g., “Not understanding other cultures”, “Cheap trips”, and “Being cheap”. Based on the images we used, the second component seems to refer to Dutch trashiness, e.g., “Loving animals”, “Going on vacation to places with many Dutch”, and “Bad taste in music”. “Boring birthdays” and “Hardworking to a fault” did not load on these components, likely because they were not recognizable to our relatively young participants. To be more specific, these depictions reflect fairly old-fashioned stereotypes about the Dutch.

Internal consistency for the first component has a Cronbach's alpha .53, and, was .29 for component 2. They were not significantly correlated, r = .55, p = .121. When we repeat the three-way analysis for both components separately, Component 1 was not significant, p = .836. However, Component 2 showed a the significant effect of self-esteem, β = -.23, t(166) = -3.17, p = .002, 95% CI [-.40, -.09], and a marginal interaction between self-esteem and national identification, β = .12, t(166) = 1.84, p = .068, 95% CI [-.01, .30], a marginal interaction between mortality salience and national identification, β = -.12, t(166) = -1.68, p = .095, 95% CI [-.29, .02], and a significant three-way interaction between mortality salience, self-esteem and national identification, β = .21, t(166) = 2.84, p = .005, 95% CI [.07, .38].

To interpret the interaction, we derived predicted means for the eight cells crossing self-esteem (± 1 *SD*) and identification (± 1 *SD*) with mortality salience condition (Aiken & West, 1991).There were no effects among participants high on national identification, β = .14, t(166) = 1.29, p = .198, 95% CI [-.08, .37]. By contrast, among participants low on national identification, there were was a main effect of self-esteem, β = -.36, t(166) = -3.62, p > .001, 95% CI [-.61, -.18], and a significant interaction between mortality salience and self-esteem, β = -.28, t(166) = --2.79, p = .006, 95% CI [-.52, -.09].

Simple slopes analyses showed that, in the control condition, self-esteem was non-significantly negatively associated with self-stereotyping among participants low on national identification, β = -.18, t(166) = -.58, p = .563, 95% CI [-.40, .22]. However, participants low on national identification showed a highly significant negative association between self-esteem and self-stereotyping in the mortality salience condition, such that participants low on national identification with low self-esteem showed more self-stereotyping, β = -.64, t(166) = -4.60, p < .001, 95% CI [-.99, -.39].

This pattern of findings further support the notion that people with national identification and low self-esteem view themselves as stereotypically Dutch under mortality salience.

Table 3. Factor analysis matrix for self-stereotyping measure

		1	2	3
1	Not understanding other cultures	.48	.14	-.13
2	Loving Animals	-.45	.52	-.04
3	Cheap trips	.71	.16	-.09
4	Going on vacation to places with many Dutch	.15	.74	.23
5	Boring birthdays	.34	.35	-.51
6	Being cheap/stingy	.77	-.01	.05
7	Bad taste in music	.27	.64	-.25
8	Hardworking to a fault	.03	.13	.88

The factor analyses take up a lot of space in the article. We therefore opted make it available as supplemental material for those interested in the “Tattas” measure.

Appendix E

Methods, Results and (short) Discussion of an Online Replication Attempt among 203 Students at the Vrije Universiteit Amsterdam

From January to October 2015, we ran an online survey (N = 203) among first-year students at the Vrije Universiteit Amsterdam. In the survey, we manipulated mortality salience and repeated key measures used in our laboratory studies. Unexpectedly, a number of notable events occurred after we started our data collection. For instance, there was a series of coordinated terrorist attacks in Paris, France that killed 130 people and injured almost 400 (Johnston, Quinn, Jalabi, & Philips, 2015, November 15) and during the unfolding European migrant crisis (Fry, 2015, September 15). From previous research, we expect news about such attacks, as well as naturally occurring factors, such as the murder of Dutch filmmaker Theo van Gogh by a radicalized Islamist, to increase prejudiced attitudes towards out-groups similar to a reminder of one's mortality, but only among those with low self-esteem (Das, Bushman, Bezemer, Kerkhof, & Vermeulen, 2009). Likewise, the negative news related to the migrant crisis may have also increased perceived out-group threat among native Dutch (Van Klingeren, Boomgaarden, Vliegenthart, & de Vreese, 2015).

Method

Participants and Design

We collected data of 264 persons. Before any data analysis, we excluded 54 non-Western participants because they were not expected to display Dutch identification. Five participants were found to have participated twice, so we excluded the data from their second participation. Two additional participants were excluded because they were not students from the Vrije

Universiteit Amsterdam. All other participants were students from this university and participated online for course credit. The final dataset consisted of 203 volunteers (179 women; M_{age} 19.87, *SD* = 3.37). They were randomly assigned to either a high (*n* = 99) versus low (*n* = 104) mortality salience condition. Self-esteem and identification were between subjects factors and key findings on out-group derogation and self-stereotyping were our main dependent variables. All participants signed an (online) informed consent form.

Materials and Measures

We measured mood (Cronbach's α T1= .81, T2 = .82), self-esteem (Cronbach's α = .90), identification (Cronbach's α = .84) and manipulated mortality salience in the same way among students at the same university as in Studies 1-2. In contrast to Studies 1-2, this study was conducted online, over a longer period of time, and students filled out the survey in their own time and setting to receive partial course credits. The participants were recruited using flyers.

To measure defensive responses after a mortality reminder, we measured participants' feelings of warmth toward Muslims, their acceptance of ethnic minorities within Dutch society (Cronbach's α = .84), and their acceptance of Muslim immigrants in Dutch society (Cronbach's α = .73) as in Study 1, as well as the ratings of the stereotypically Dutch images (Cronbach's α = .87), and the measure of group-stereotyping (Cronbach's α = .73) and self-stereotyping (Cronbach's α = .68) as in Study 2.

Results

Self-esteem and ethic identification. On the 7-point scale, our sample reported a mean self-esteem of 5.14 (*SD* = 1.01) and a mean ethnic identification of 5.81 (*SD* = 1.10). Self-esteem and identification were significantly correlated in this sample, $r(203) = .18$, $p = .011$.

Mood. We conducted a repeated measures ANOVA with mood as within-subject factor and mortality salience as between-subject factor. There was no interaction effect between mood and mortality salience, $F(1,201) = .45$, $p < .503$. When we added self-esteem as a covariate, we found a main effect, such that negative mood decreased from Time 1 ($M = 2.65$, $SD = .83$) to Time 2 ($M = 2.61$, $SD = .91$), but also an interaction between self-esteem and change in negative mood, $F(1,201) = 6.72$, $p = .010$, $\eta_p^2 = .03$, such that self-esteem was associated with a stronger decrease in negative mood as in Studies 1-2.

Warmth ratings for Muslims. The three-way interaction between mortality salience, self-esteem, and identification was not significant, $\beta = -.004$, $t(194) = -.05$, $p = .963$, 95% CI [-3.64, 3.47]. We found a weak trend suggesting a two-way interaction between mortality salience and self-esteem, $\beta = .112$, $t(194) = 1.59$, $p = .114$, 95% CI [-.67, 6.17]. Simple-slopes analyses showed that under mortality salience, low self-esteem was marginally associated with decreased feelings of warmth towards Muslims, $\beta = .20$ $t(194) = 1.88$, $p = .061$, 95% CI [-.23, 9.88]. Under dentist salience, self-esteem had no influence, $\beta = -.03$, $t(194) = -.29$, $p = .773$, 95% CI [-5.28, 3.93]. The warmth ratings for the other groups were not significant, *t*s < -1.08, *p*s > .281.

Figure E1. Warmth ratings for Muslims as a function of self-esteem and mortality salience. Low and high levels of self-esteem were coded as 1 *SD* below and above the mean. Feelings of warmth on the thermometer were rated on a scale from 0, *very cold or negative feelings*, to 100, *very warm and positive feelings.*

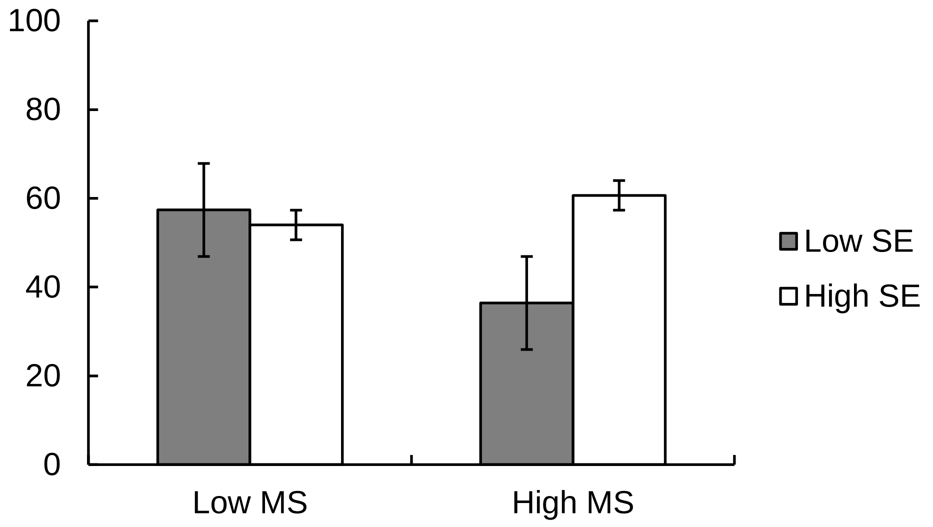

Acceptance of ethnic minorities. The three-way interaction between mortality salience, self-esteem, and identification was not significant, β = .04, t(194) = .44, p = .663, 95% CI [-.10, .16]. However, we found a two-way interaction between mortality salience and self-esteem, β = .184, t(194) = 2.62, p = .009, 95% CI [.04, .29]. Simple-slopes analyses showed that under mortality salience, low self-esteem was associated with decreased acceptance of ethnic minorities, β = .27 t(194) = 2.731, p = .015, 95% CI [.54, .41]. Under dentist salience, self-esteem had no influence, β = -.11, t(194) = -1.20, p = .233, 95% CI [-.27, .07].

Figure E2. Acceptance of ethnic minorities as a function of self-esteem and mortality salience. Low and high levels of self-esteem were coded as 1 *SD* below and above the mean, respectively. The items were rated on a scale from 1, *not at all*, to 7, *very much*.

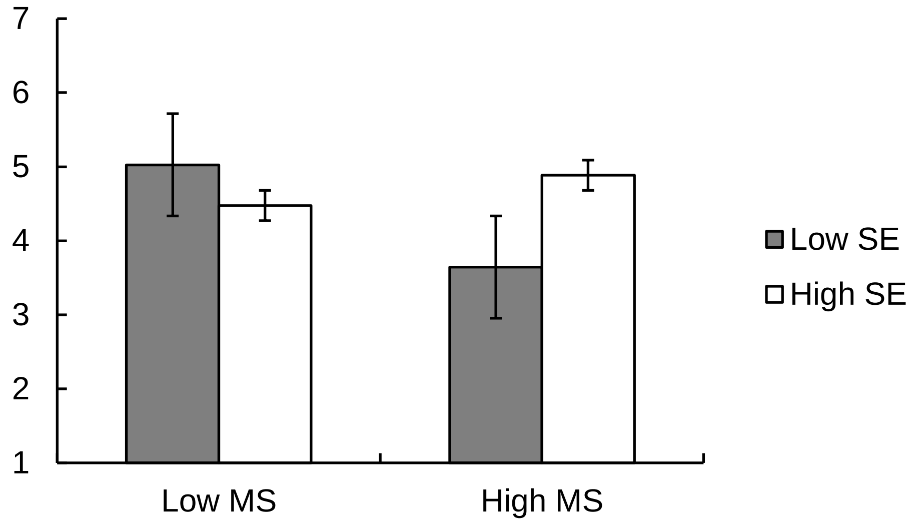

Acceptance of Muslim Immigrants in Dutch society. The three-way interaction between mortality salience, self-esteem, and identification was not significant, β = .03, t(194) = .41, p = .681, 95% CI [-.14, .21]. However, leaving identification out of the analysis, we found a main effect of self-esteem, β = .19, t(198) = 1.69, p = .050, 95% CI [.00, .33], and a two-way interaction between mortality salience and self-esteem, β = .19, t(198) = 2.69, p = .008, 95% CI [.06, .39]. Simple-slopes analyses showed that under mortality salience, lower self-esteem was associated with decreased acceptance towards Muslims, β = .32 t(198) = 3.15, p = .002, 95% CI [.15, .63]. Under dentist salience, self-esteem had no influence, β = -.05, t(198) = -.535, p = .593, 95% CI [-.28, .16].

Figure E3. Acceptance of Muslim immigrants in Dutch society as a function of self-esteem and mortality salience. Low and high levels of self-esteem were coded as 1 *SD* below and above the mean, respectively. Items were rated on a scale from 1, *not at all,* to 7, *very much*.

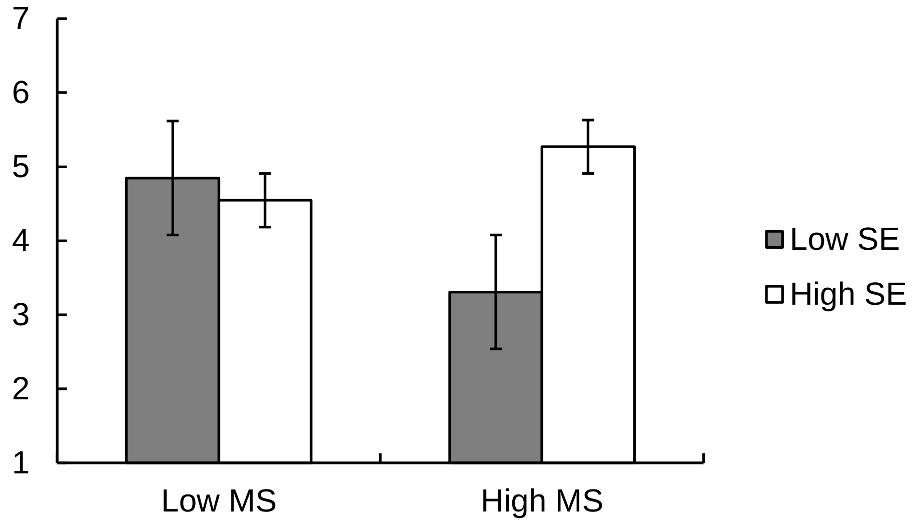

Appreciation of humorous depictions of negative Dutch stereotypes. On average, participants rated the images between 2.34 (*SD* = 1.52) and 4.97 (*SD* = 1.78) on how funny they were, similar to Study 2. These were the overall mean ratings of how funny, hurtful and insulting the images were rated: *M* = 4.01 (*SD* = 1.50), *M* = 2.76 (*SD* = 1.58), and *M* = 3.08 (*SD* = 1.61).

The three-way interaction between mortality salience, self-esteem, and identification was not significant, *β* = .009, *t*(195) = .11, *p* = .914, 95% CI [-.14, .16]. However, leaving identification out of the analysis yielded a marginally significant interaction between mortality salience and self-esteem, *β* = -.12, *t*(199) = -1.79, *p* = .076, 95% CI [-.27, .01]. Simple-slopes analyses showed that under low threat, individuals with low self-esteem rated the images as less funny, *β* = .20, *t*(199) = 2.17, *p* = .032, 95% CI [.02, .41]. However, in the high threat condition, self-esteem had no effect, *β* = -.05 *t*(199) = -.44, *p* = .659, 95% CI [-.26, .17].

Figure E4. Appreciation of humorous depictions of negative Dutch stereotypes as a function of self-esteem and mortality salience. Low and high levels of self-esteem were coded as 1 *SD* below and above the mean, respectively. Items were rated on a scale from 1, *not at all,* to 7, *very much*.

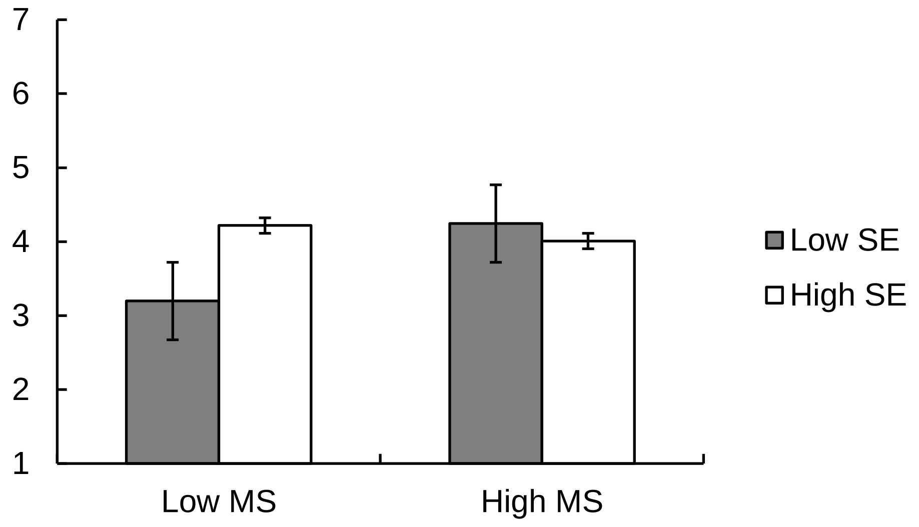

Group stereotyping. There were no effects on how much participants stereotyped their own people, *ts* < -1.14, *ps* > .254.

Self-stereotyping. The three-way interaction between mortality salience, self-esteem, and identification was not significant, β = .01, *t*(195) = .167, *p* = .867. However, leaving identification out of the analysis yielded a marginally significant interaction between mortality salience and self-esteem, β = -.13, *t*(199) = -1.95, *p* = .053, 95% CI [-.24, .002]. Simple-slopes analyses showed that under mortality salience, low self-esteem was associated with increased self-stereotyping, β = -.41 *t*(199) = -4.05, *p* < .001, 95% CI [-.54, -.19]. Under dentist salience, self-esteem was not associated with self-stereotyping, β = -.14, *t*(199) = -1.55, *p* = .122, 95% CI [-.29, .03].

Figure E5. Self-stereotyping as a function of self-esteem and mortality salience. Low and high levels of self-esteem were coded as 1 *SD* below and above the mean, respectively. Items were rated on a scale from 1, *not at all,* to 7, *very much*.

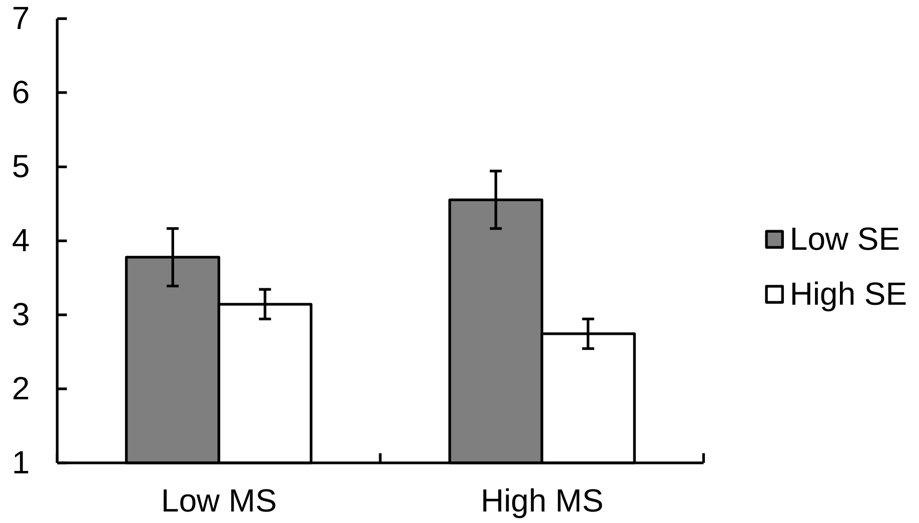

Table E1. Means and standard deviations of the ratings of the stereotypically Dutch images.

	Description of stereotypical depiction and accompanying caption	*M*	*SD*
1	Man and woman dancing at a club with the caption ‘Hardcore is the soundtrack of our lives’	3.65	1.93
2	Person lying in a hospital bed completely covered in bandages with the caption ‘You are still coming to work, right?’	4.39	1.86
3	Birthday celebration where all the guests sit in the living room with the caption ‘Happy birthday with Els’ repeated many times	4.97	1.78
4	Man and woman sitting at a camping site with their trailer with the caption ‘We are truly nature people’	4.48	1.67
5	Woman being overly excited about free ‘liverwurst’ a popular type of sausage with the caption ‘Well, I won’t say no to that!’	4.82	1.52
6	Man and woman on a date with the caption ‘Can you transfer half of the check back to my bank account?’	4.49	1.77
7	A big discount sale of a popular Dutch chain store with the caption ‘Family daytrip’	3.81	1.86
8	Dutch men at a Dutch fast food place in Crete (a popular Dutch travel destination) with the caption ‘Having a cultural experience on Crete’	4.94	1.59
9	Sandwiches with cheese with the caption ‘Day trip to the amusement park’	4.65	1.69
10	Dog licking a woman on the mouth with the caption ‘Oh it’s just my dog!’	2.92	1.89
11	A Dutch man talking to an African-American man with the caption ‘Racism only exists because you are still fussing about it’	2.34	1.52
12	Dutch person touching an African-American woman’s hair with the caption ‘May I touch it’	3.93	1.74
13	An elderly Dutch lady with the caption ‘Do you speak Dutch or ‘Muslim’ at home?’	3.55	1.94
14	An elderly Dutch lady saying with the caption ‘Oh, you’re Moroccan. What a coincidence! My neighbor is Turkish.’	3.19	1.82

15	A Dutch man talking to an African-American woman with the caption ‘I face racism all the time because of my French last name’	3.13	1.83

Table E2. Means and standard deviations of the ratings of how typical these items are of Dutch people and of themselves.

	Typical of Dutch		Typical of self	
Item	*M*	*SD*	*M*	*SD*
Not understanding other cultures	4.00	1.75	2.26	1.31
Loving animals	4.05	1.48	4.51	1.98
Cheap trips	4.95	1.54	3.70	1.74
Going on vacation to places with many Dutch	5.45	1.31	2.77	1.78
Boring birthdays	4.19	1.82	2.94	1.67
Being cheap/stingy	5.24	1.32	3.45	1.61
Bad taste in music	3.40	1.73	2.21	1.31
Hardworking to a fault	5.07	1.18	5.30	1.27

Discussion

We found no reliable effects of ethnic identification in this online study. There were a couple notable changes compared to the lab studies. First, we collected the data over a longer period of time among first-year students who participated in their own time, and at a time and location of their own choice for course credit. Secondly, self-esteem and ethnic identification were positively correlated in this sample. Nonetheless, we found that mortality salience decreased acceptance of ethnic minorities and Muslim immigrants in Dutch society among people with low self-esteem. Moreover, people with low self-esteem found stereotypical images about the Dutch people funnier and showed increased self-stereotyping in the mortality salience condition.

We tentatively suggest that events that concern all Dutch citizens may have increased the salience of the Dutch identity in this sample. Specifically, the media reports on the terrorist

attacks in Paris (Das et al., 2009; Jaspal & Cinnerella, 2010) and the threat of a loss of resources and employment opportunities associated with the influx of migrants (Stephan, Ybarra, & Morrison, 2009) may have led Dutch citizens to categorize themselves as a more uniform group (Hogg & Turner, 1987). Increased self-stereotyping is one indication that people were indeed defining themselves through their ethnic identity.

In line with this reasoning, previous research has shown that people who were reminded of their mortality showed increased endorsement of in-group values and rejection of out-group values depending on the social identity that was made salient at the time (Halloran, & Kashima, 2004; Giannakakis, & Fritsche, 2011). For instance, Americans who were reminded of 9/11, a foreign threat that concerned all American citizens, reported increased national identification compared to when they were reminded of a domestic threat (Davies, Steel, & Markus, 2008). Thus, salient events in the larger societal context may strongly influence people's perceived belonging to, and also their perceived ability to distance from, relevant in-groups, and thereby their defensive responses.

.

References

Aiken, L.S., & West, S.G. (1991). *Multiple regression: Testing and interpreting interactions*. Newbury Park, CA: Sage Publications.

Das, E., Bushman, B. J., Bezemer, M. D., Kerkhof, P., & Vermeulen, I. E. (2009). How terrorism news reports increase prejudice against outgroups: A terror management account. *Journal of Experimental Social Psychology, 45,* 453-459. doi:10.1016/j.jesp.2008.12.001

Pratto, F., Çidam, A., Stewart, A. L., Zeineddine, F. B., Aranda, M., Aiello, A., et al. (2013). Social dominance in context and in individuals: Contextual moderation of robust effects of Social Dominance Orientation in 15 languages and 20 countries. *Social Psychological and Personality Science, 4*(5), 587-59. doi:10.1177/1948550612473663

van Klingeren, M., Boomgaarden, H. G., Vliegenthart, R., & de Vreese, C. H. (2015). Real world is not enough: the media as an additional source of negative attitudes toward immigration, comparing Denmark and the Netherlands. *European Sociological Review, 31* (3), 268-283. doi:10.1093/esr/jcu089
